# Age and Gender Differences in Urinary Levels of Eleven Phthalate Metabolites in General Taiwanese Population after a DEHP Episode

**DOI:** 10.1371/journal.pone.0133782

**Published:** 2015-07-24

**Authors:** Po-Chin Huang, Chih-Hsin Tsai, Wei-Yen Liang, Sih-Syuan Li, Wen-Harn Pan, Hung-Che Chiang

**Affiliations:** 1 National Environmental Health Research Center, National Institute of Environmental Health Sciences, National Health Research Institutes, Miaoli, Taiwan; 2 Institute of Biomedical Sciences, Academia Sinica, Taipei, Taiwan; 3 Division of Preventive Medicine and Health Service Research, Institute of Population Health Sciences, National Health Research Institutes, Miaoli, Taiwan; 4 Division of Environmental Health and Occupational Medicine, National Institute of Environmental Health Sciences, National Health Research Institutes, Miaoli, Taiwan; Qingdao Agricultural University, CHINA

## Abstract

**Introduction:**

In 2011, the Taiwan FDA disclosed illegal di(2-ethylhexyl phthalate) (DEHP) and dibutyl phthalate (DBP) use in beverage and nutrition supplements. We aim to determine phthalate exposure and other relevant factors in a sample of the general Taiwanese population in order to evaluate actual phthalate exposure levels after this disclosure of DEHP use.

**Method:**

We selected subjects aged 7 years old and older in 2013 from the general Taiwanese population. First morning urine samples from each participant were collected to analyze 11 phthalate metabolites representing 7 parent phthalates using on-line liquid chromatography/ tandem mass spectrometry. An interview questionnaire was applied to obtain participant demographic characteristics, lifestyle, and other relevant factors.

**Results:**

The median levels of metabolites of DEHP, including mono-ethylhexyl phthalate (MEHP), mono-(2-ethyl-5-oxohexyl) phthalate (MEOHP), mono-(2-ethyl-5-hydroxyhexyl) phthalate (MEHHP), mono-(2-ethyl-5-carboxypentyl) phthalate (MECPP), DBP (DnBP and DiBP), including mono-n-butyl phthalate (MnBP) and mono-iso-butyl phthalate (MiBP), and mono-ethyl phthalate (MEP) in urine samples of 290 adults/ 97 minors (<18 years) were 7.9/ 6.1, 12.6/ 17.8, 22.0/ 25.8, 25.4/ 30.8, 18.1/ 23.6, 9.4/ 13.6 and 14.5/ 12.4 μg/g creatinine, respectively. Women (≧18 years) were exposed to significantly higher levels of MEHHP (P=0.011), MECPP (P=0.01), MnBP (P=0.001) and MEP (P<0.001) than men (≧18 years), whereas no gender difference was observed in minors. We found significant higher level of MEP (creatinine-unadjusted) in subject aged between 18 to 40 years old (P<0.001), especially for women. Exposure levels of MEOHP (P<0.001), MECPP (P=0.002) and MnBP (P=0.044) in minors were significantly higher than those of adults. High frequency usage of food preservation film and bags, and personal care products are potential sources of phthalates exposure in general Taiwanese.

**Conclusion:**

Our findings indicated that DEHP and DBP exposure in a sample of the general Taiwanese population varied by age and gender, possibly affected by different lifestyles, and continuing bio-monitoring surveillance is warranted.

## Introduction

Phthalates are widely used in many daily products such as di-2-ethylhexyl phthalate (DEHP) in plastics, toys and medical equipment; diethyl phthalates (DEP) in cosmetics and personal care products; and di-*n*-butyl phthalate (DnBP) in food package film and plastic products (S1 Table). Humans may be exposed to phthalates mainly through ingestion of phthalate-tainted food, or inhalation and dermal absorption of phthalate-contained products; urinary metabolites are considered to be good biomarkers for assessing their exposure [1–2]. In 2011, the Taiwan Food and Drug Administration discovered illegal-use of phthalates, such as DEHP, DnBP and di-i-butyl phthalate (DiBP) in beverage, food and nutrition supplements [3–4]. Although most contaminant products were removed and immediate regulatory action occurred, human phthalate exposure monitoring remains necessary to evaluate actual phthalate exposure levels and their potential risk in the Taiwanese population.

Growing evidence has indicated some endocrine-related effects of phthalates on human health [5], especially in the thyroid and reproductive glands [6]. Some studies have revealed that phthalates may decrease thyroid and growth hormones in different races [7–10]. Other studies have revealed concerns related to phthalate exposure effects on pediatric allergy diseases [11–12] and adult infertility [13–15]. Some studies have indicated that several factors, such as age, gender, or lifestyle, may affect the exposure levels of phthalates [16–18] and influence their effects on the health of different populations.

A well-organized human biomonitoring study is useful for determining the exposure dose in the general population and assessing exposure, risk, and management of complications [19]. Some studies, including the German Environmental Survey (GerEs) [19] and National Health and Nutrition Survey (NHANES) [2], have demonstrated the time-trend variation and potential influence of environmental chemicals in the general population. Recent studies, such as the Canadian Health Measures Survey (CHMS) [20], Korean Environmental Health Survey (KEHS) [21], Consortium to Perform Human Biomonitoring on a European Scale (COPHES) and DEMOCOPHES study [22], Belgium (FLEHS) [23] and Danish studies [24], have revealed exposure profiles of populations affected by environmental chemicals differ according to countries, lifestyles, and exposure sources. We aimed to conduct an exposure assessment study of phthalates, a part of the Taiwan Environmental Surveys for Toxicants (TESTs), using biosamples from a general population in Taiwan and evaluating the effects of relevant factors such as demographic characteristics and lifestyle on phthalate exposure.

## Materials and Methods

### Ethics statement

This study was approved by Research Ethics Committee, National Health Research Institutes (No. EC1020206) in Taiwan. Written informed consent from each participant and additional signatures from their parents for minors was obtained prior to study enrollment.

### Subject recruitment and data collection

**Fig 1:**
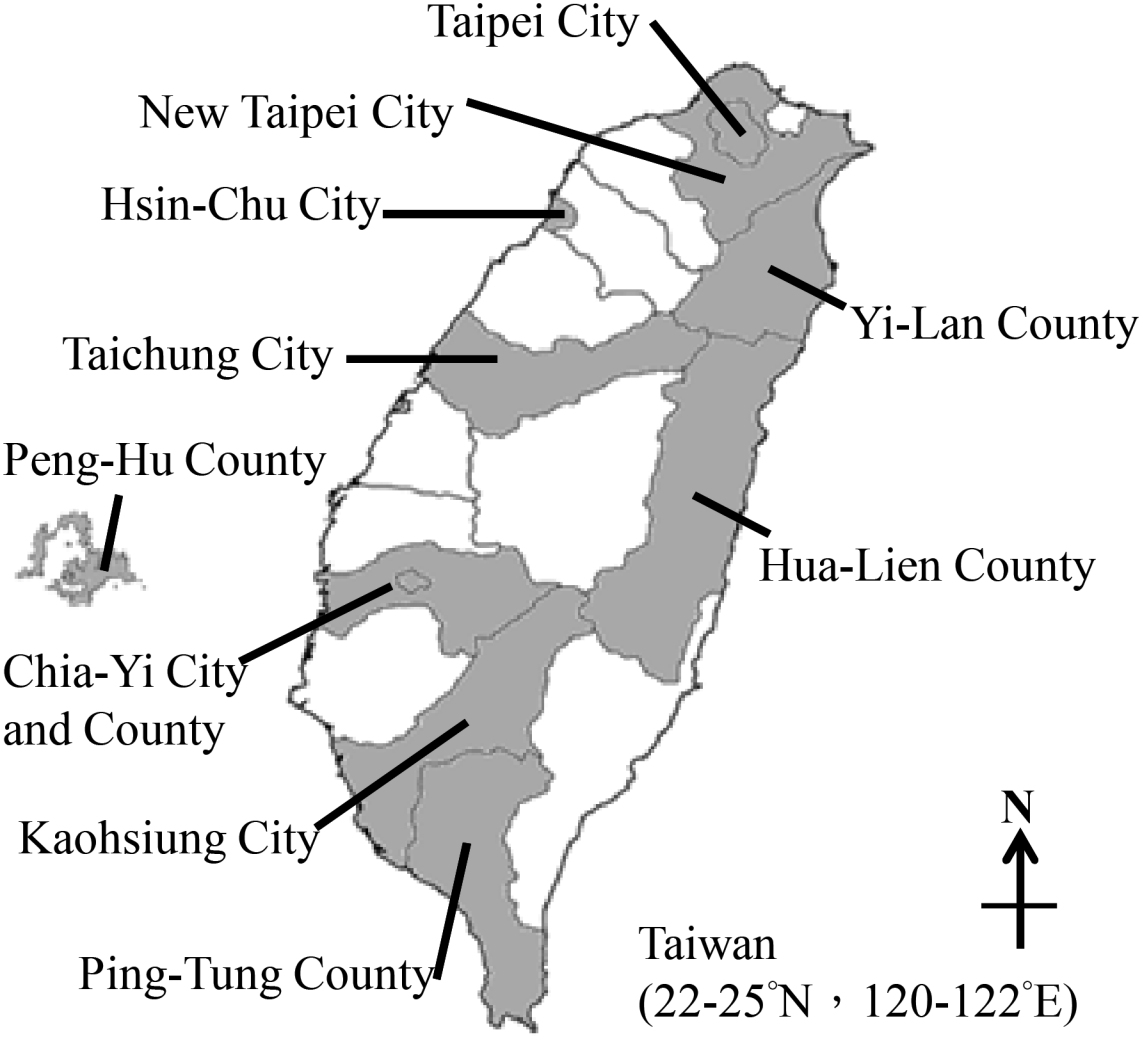
Eleven sampling sites of the TEST study in Taiwan.

In order to obtain sufficient participation from the general Taiwanese population, we collaborated with the National and Nutrition Health Survey in Taiwan (NAHSIT) team and used the same sampling frame and procedure in subject recruitment [25]. There are five major regions and 22 cities/ counties in Taiwan (Fig 1). Briefly, NAHSIT selected twenty major cities/ counties of Taiwan and selected subjects in all age groups. According to the population density and urbanization of each city in Taiwan, each city township is classified into two groups. Then, NAHSIT randomly selected two townships of each group to represent a city/ county. Subjects had to be Taiwanese ≥7 years old, and excluded pregnant and breast-feeding women, individuals with severe disease (e.g. cancer patients), foreigners, and citizens in hospitals and jails [25]. We selected seventeen townships of eleven cities/ counties in the northern region (ex.: Taipei and New Taipei City), central region (ex.: Taichung and Chia-Yi City), southern region (ex.: Kaohsiung City), eastern region (ex.: Hua-Lien County), and the remote island region (Peng-Hu County) of Taiwan (Fig 1) during a time period from May to December 2013. We interviewed a total of 500 subjects on the day of health examination at community center or elementary school; 394 subjects participated in this study, which yielded a response rate around 78%. Most of the subjects who refused participation were children afraid to provide samples.

After informed consent was obtained, first morning urine samples (20 mL) were collected using a PP container and transferred into an acetonitrile (ACN)-prewashed amber glass bottle and stored at -20 ℃. An interview questionnaire was also used to obtain information about participant demographics (age, gender, residence, education), environmental exposure (cigarette smoking, pesticide usage), lifestyles (plastics product usage, personal care products), and other related factors (disease history).

### Phthalate metabolite analysis

Eleven phthalate metabolites including mono-ethylhexyl phthalate (MEHP), mono-(2-ethyl-5-oxo-hexyl) phthalate (MEOHP), mono-(2-ethyl-5-hydroxyhexyl) phthalate (MEHHP), mono-(2-ethyl-5-carboxypentyl) phthalate (MECPP), mono-(2-carboxymethylhexyl) phthalate (MCMHP), mono-*n*-butyl phthalate (MnBP), mono-iso-butyl phthalate (MiBP), mono-ethyl phthalate (MEP), mono-iso-nonyl phthalate (MiNP), mono-benzyl phthalate (MBzP) and mono-methyl phthalate (MMP) representing exposure to seven commonly used phthalates (DEHP, DnBP, DiBP, DEP, di-iso-nonyl phthalate (DiNP), benzyl butyl phthalates (BBzP) and dimethyl phthalate (DMP) were measured in each urine sample. We used a modified method previously described by Koch et al. [26] using on-line chromatography to increase the efficiency and accuracy of analysis. After a urine sample was thawed and sonicated for 10–15 min, the urine sample (100 μl) was loaded into a glass vial (2 ml) which contained ammonium acetate (AA, 20 μl, >98%, Sigma Aldrich Lab., Inc., St. Louis, MO, USA), β-glucuronidase (10 μl, E.coli K12, Roche Biomedical, Mannheim, Germany), and a mixture of ten isotopic (^13^C_4_) phthalate metabolite standards (100 μl. Cambridge Isotope Lab., Inc., Andover, MA, USA). After the sample was incubated (37℃, 90 min), a 270 μl solution (5% ACN), Merck, Darmstadt, Germany) with 0.1% formic acid (FA, Merck, Darmstadt, Germany) was added and sealed with the PTEF cap for analysis. We used an on-line system which was coupled with liquid chromatography/electrospray tandem mass spectrometry (LC–ESI-MS/MS) (Agilent 1200/ API 4000, Applied Biosystems, Foster City, CA, USA). We used two columns in our on-line system. One C18 column (Inertsil ODS-3, 33×4.6 mm, 5 μm, GL Science, Tokyo, Japan) was used to extract and clean our sample, and an analytical column (Inertsil Ph, 150 *4.6 mm, 5 μm, GL Science, Tokyo, Japan) was used to separate different phthalate metabolites. The gradient program of the clean-up column was listed as follows: 100% solution A (5% ACN + 0.1% FA) (0–7 min), 100% solution B (90% ACN + 0.1% FA) (7–9 min), 100% solution A (9–10 min) and continued to 12 min. The flow rate was set at 1000 μl/min. The analytical column gradient program was listed as follows: 100% solution C (50% ACN + 10 mmole AA) (0–3.6 min), 100% solution D (95% ACN + 10 mmole AA) (3.6–8.6 min), 100% solution C (8.6–9 min) and continued to 12 min. We used a negative multiple reaction monitoring model for mass spectroscopy detection. The ion pair of each phthalate metabolites was listed as follows: MEHP (277/134), MEOHP (291/143), MEHHP (293/121), MECPP (307/159), MCMHP (307/113), MnBP (221/71), MiBP (221/71), MEP (193/121), MiNP (291/121), MBzP (255/183), and MMP (179/107). The detection limits of MEHP, MEOHP, MEHHP, MECPP, MCMHP, MnBP, MiBP, MEP, MiNP, MBzP, and MMP were 0.7, 0.3, 0.3, 0.3, 0.1, 1, 1, 0.3, 0.1, 0.3 and 0.3 ng/mL respectively. One blank, repeat, and quality control (QC) sample were included in each batch of analyzed samples. The concentration of blank samples shall be below two times the detection limit. The QC sample was spiked in a pooled urine sample with a mixture of phthalate metabolite standards (20–50 ng/ml) in each sample. The relative percent difference of repeat sample and recovery of QC sample shall be within ± 30%. Urinary creatinine levels were measured by spectrophotometric methods, with picric acid as a reactive compound for reading at 520 nm measurement (Beckman; DXC 800).

### Statistical Analysis

Levels of urinary phthalate metabolites and creatinine were evaluated as continuous variables. Meanwhile, age, gender, residence, education, family income, cigarette smoking and beverage status (tea, coffee, alcohol), as well as the frequency of plastic products, personal care products, and pesticide use were evaluated as nominal variables. All measured phthalate metabolites were logarithmically (log_10_) transformed for Pearson correlation analysis. We categorized our subjects into adults (≧18 years old) and minors (<18 years old), and further stratified four comparatively sized age groups: ≧7~<18 years old, ≧18~<40 years old, ≧40~<65 years old, ≧65 years old (elders). The Mann-Whitney U test, Chi-square test, and Kruskal-Wallis test were used to evaluate differences between demographic data, such as age and gender, region, and each level of phthalate metabolites for continuous and categorical variables, respectively. We calculated not detectable (ND) levels, below the detection limit, as 1/2 detection limit of each phthalate metabolite, and detectable rate as number of urine sample with level of each phthalate metabolite above detection limit divided by all analyzed urine samples. We presented our data both in a creatinine-based level, which adjusted for individual urinary excretion rate, and creatinine-unadjusted level in the parentheses. Pearson correlation coefficients assessed correlations between age and each urinary phthalate metabolite level. Commercially available statistical software (SPSS version 22.0; SAS Institute, Cary, NC, USA) was used for all statistical analysis. The level of significance was set at *P*< 0.05.

## Results

### Characteristics of Subjects

A total of 296 adults and 98 minors with completed data were included for statistical analysis in this study. In adults, most participants were between 40–65 years of age (44%), married (72.9%), well-educated (56.8%), middle-class status (57%), non-smokers (74.3%), and reported no pesticide use at home (74.7%); around half were tea and coffee drinkers (57.4% and 42.9%, respectively). A high percentage of participants did not drink alcohol (85.8%) or chew betel nuts (93.2%). For minors, around half of them were boys (55%), below 12 years old (52%); half of them reported as tea drinkers and passive smokers. The detailed demographic characteristics of all participants were shown in Table 1.

**Table 1:** Demographic characteristics of all participants in this study (N = 394).

Characteristics	Item	Adults (≧18 years, n = 296)		Minors (<18 years, n = 98)	
		n	%	n	%
Gender	Male	136	45.9	54	55.1
	Female	160	54.1	44	44.9
Age (years)	18–40/ 7–12	71	24.0	51	52.0
	40–65/ 12–18	131	44.2	47	48.0
	65 and older	94	31.8	0	0
Region	Northern Taiwan	90	30.4	31	31.6
	Central Taiwan	39	13.2	15	15.3
	Southern Taiwan	82	27.7	24	24.5
	Eastern Taiwan	56	18.9	12	12.2
	Remote island	29	9.8	16	16.4
Marriage status	Single	49	16.6	97	99.0
	Married	216	72.9	1	1.0
	Divorce/ widowed	31	10.5	0	0
Education	≦Elementary school	84	28.3	51	52.0
	Junior high school	44	14.9	29	29.6
	Senior high school	68	23.0	18	18.4
	≧College/ Graduates	100	33.8	0	0
Annual family income ^a^	<15,625	169	59.5	39	43.3
	15,625~31,250	74	26.1	30	33.3
	>31,250	41	14.4	21	23.4
Cigarette smoking ^b^	Yes/ No	75/ 220	25.4/ 74.6	2/ 96	2.0/ 98.0
Passive smoker	Yes/ No	148/ 147	50.2/ 49.8	50/ 47	51.4/ 48.6
Alcohol consumption ^c^	Yes/ No	36/ 255	12.4/ 87.6	1/ 96	1.0/ 99.0
Tea drinking ^d^	Yes/ No	171/ 124	58.0/ 42.0	49/ 49	50.0/ 50.0
Coffee drinking ^d^	Yes/ No	127/ 169	42.9/ 57.1	6/ 92	6.1/ 93.9
Betel nut chewing ^e^	Yes/ No	20/ 276	6.8/ 93.2	1/ 97	1.0/ 99.0
Pesticide use at home	Yes/ No	75/ 221	25.3/ 74.7	28/ 70	28.6/ 71.4

^a^ The currency exchange rate of converting USD to new Taiwan dollar is 1:32.

^b^ Subjects who self-reported consuming at least one cigarette per day.

^c^ Subject who self-reported consuming at least one bottle of alcohol drink per week.

^d^ Subjects who self-reported consuming at least one cup of tea or coffee per week.

^e^ Subject who self-reported chewing at least one betel nut per week.

### Levels of eleven phthalate metabolites

**Fig 2:**
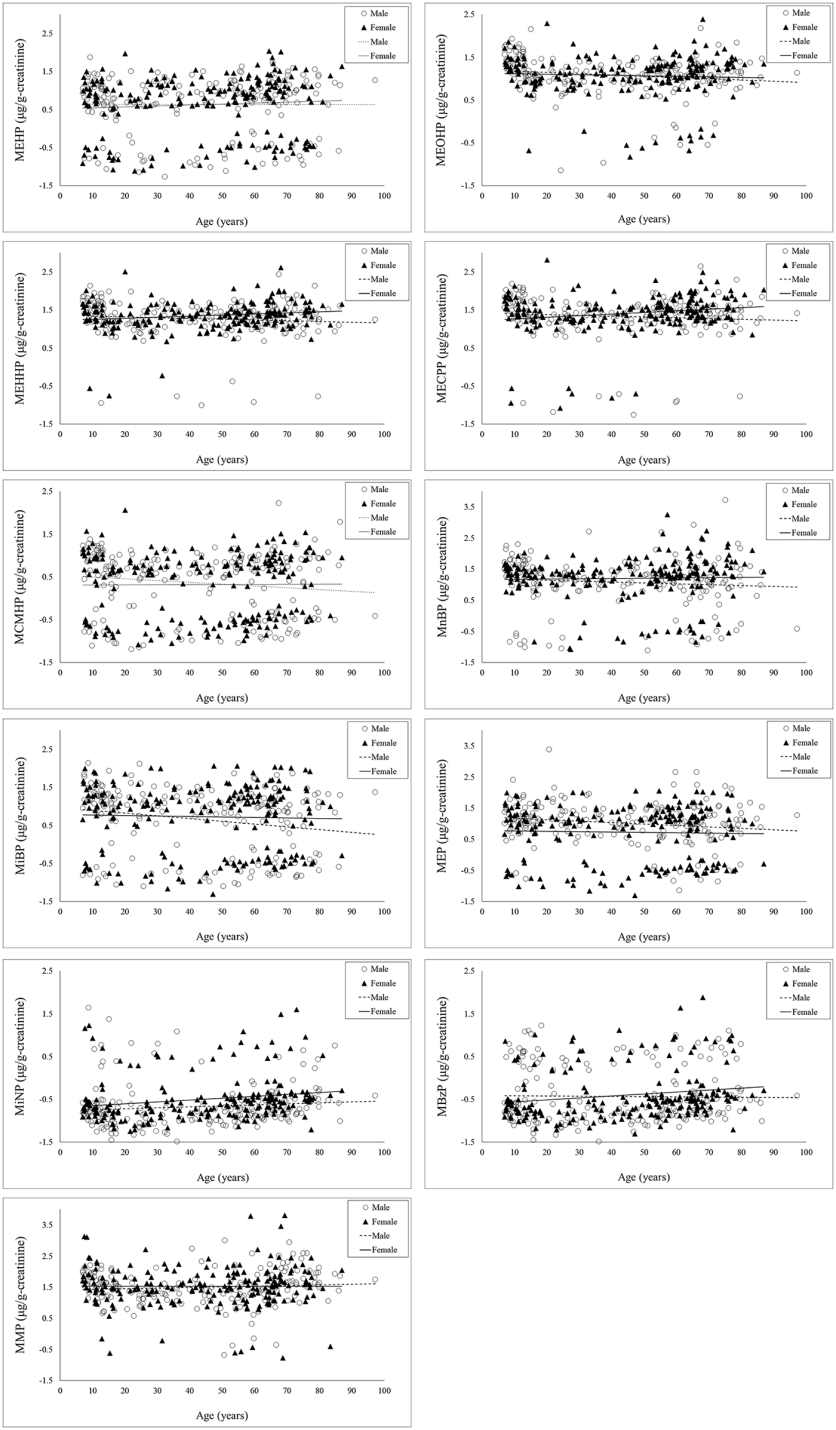
Distribution of 11 phthalate metabolites in a sample of the general Taiwanese population (N = 387) ≥7 years of age.

Fig 2 and Table 2 (un-adjusted data was shown in the parentheses of Table 2) showed the distribution and creatinine-adjusted concentrations of eleven phthalate metabolites in our participants classified by age and gender, respectively. For adults, the median levels of MEHHP (women/ men: 24.1 vs. 18.3 μg/g-c, *P* = 0.011), MECPP (28.3 vs. 24.4 μg/g-c, *P* = 0.010), MnBP (22.1 vs. 13.8 μg/g-c, *P* = 0.001), MEP (19.6 vs. 10.4 μg/g creatinine [μg/g-c], *P*<0.001), MBzP (0.3 vs. 0.2 μg/g-c, *P<*0.001), MiNP (0.3 vs. 0.2 μg/g-c, *P<*0.001) in women (≧18 year old, n = 156) were significantly higher than those seen in men (≧18 year old, n = 134). For minors, the median levels of most phthalate metabolites in boys (n = 54) was higher than those in girls (n = 43), but no statistical significance was observed. We found that the median levels of MEOHP, MEHHP, MECPP, MnBP, MiBP and MMP in minors were higher than those seen in adults.

**Table 2:** Distribution of creatinine-adjusted levels (μg/g creatinine or ng/ml) of phthalate metabolites ^a^ in a sample of the general Taiwanese population (N = 387) by minors and adults.

Phthalate metabolites	Group	Detectable rate (%)	n	GM		Min		5th		25th		50th		75th		95th		Max		*P*-value ^b^	
MEHP	***Adults***	76.4	290	4.4	(3.4)	ND	(ND)	ND	(ND)	2.4	(2.1)	7.9	(6.6)	15.5	(12.2)	34.9	(30.5)	110.3	(138.9)	0.211	(0.556)
	Men	75.7	134	4.0	(3.6)	ND	(ND)	ND	(ND)	1.9	(1.6)	7.7	(7.1)	15.0	(13.9)	31.9	(37.7)	51.0	(62.9)	0.414	(0.215)
	Women	76.9	156	4.8	(3.3)	ND	(ND)	ND	(ND)	3.9	(2.5)	8.0	(6.1)	15.9	(11.3)	40.7	(28.6)	110.3	(138.9)		
	***Minors***	80.6	97	4.0	(4.1)	ND	(ND)	ND	(ND)	2.4	(3.1)	6.1	(7.2)	12.5	(12.2)	27.2	(27.5)	74.3	(69.1)		
	Boys	88.9	54	5.2	(6.3)	ND	(ND)	ND	(ND)	3.2	(4.6)	6.6	(8.6)	13.8	(15.7)	27.3	(36.5)	74.3	(69.1)	0.196	(0.009)
	Girls	70.5	43	2.8	(2.4)	ND	(ND)	ND	(ND)	ND	(ND)	5.3	(5.9)	11.2	(8.7)	30.0	(24.8)	37.2	(27.1)		
MEOHP	***Adults***	93.2	290	10.7	(8.3)	ND	(ND)	ND	(ND)	8.2	(5.6)	12.6	(10.3)	20.2	(16.5)	38.2	(33.7)	244.5	(289.6)	<0.001	(<0.001)
	Men	94.1	134	10.0	(9.1)	ND	(ND)	ND	(ND)	7.5	(5.9)	12.0	(11.5)	18.3	(18.5)	29.7	(35.9)	146.5	(178.8)	0.091	(0.115)
	Women	92.5	156	11.3	(7.6)	ND	(ND)	ND	(ND)	8.4	(5.5)	13.6	(9.6)	21.6	(14.9)	43.6	(33.9)	244.5	(289.6)		
	***Minors***	99.0	97	16.9	(17.2)	ND	(ND)	4.3	(4.2)	9.6	(9.7)	17.8	(19.1)	29.1	(31.3)	61.5	(80.7)	140.2	(150.0)		
	Boys	100	54	19.3	(23.0)	3.4	(3.5)	5.1	(5.2)	11.3	(15.0)	19.8	(24.4)	36.3	(33.6)	68.7	(108.5)	140.2	(150.0)	0.159	(0.001)
	Girls	97.7	43	14.2	(11.9)	ND	(ND)	4.2	(2.5)	8.7	(6.6)	16.2	(15.1)	25.4	(21.1)	54.9	(59.4)	72.8	(68.9)		
MEHHP	***Adults***	98.0	290	20.2	(15.6)	ND	(ND)	6.3	(4.1)	13.4	(9.8)	22.0	(16.3)	32.6	(30.0)	73.6	(69.0)	415.7	(487.7)	0.116	(<0.001)
	Men	96.3	134	16.8	(15.3)	ND	(ND)	4.7	(3.1)	11.6	(8.9)	18.3	(18.2)	29.3	(31.5)	58.2	(75.3)	266.7	(325.4)	0.011	(0.425)
	Women	99.4	156	23.6	(15.9)	ND	(ND)	7.3	(4.8)	14.7	(9.9)	24.1	(15.1)	33.7	(25.7)	87.6	(61.8)	415.7	(487.7)		
	***Minors***	96.9	97	21.9	(22.3)	ND	(ND)	6.2	(6.4)	14.9	(14.6)	25.8	(25.1)	43.9	(38.7)	84.7	(104.4)	134.7	(245.9)		
	Boys	98.1	54	25.0	(29.8)	ND	(ND)	6.2	(6.7)	14.8	(19.3)	26.8	(29.2)	50.3	(57.3)	89.0	(162.1)	134.7	(245.9)	0.153	(0.002)
	Girls	95.5	43	18.5	(15.5)	ND	(ND)	1.8	(0.8)	14.8	(10.0)	19.1	(20.0)	37.6	(28.3)	69.9	(64.0)	101.3	(91.0)		
MECPP	***Adults***	95.9	290	22.3	(17.2)	ND	(ND)	6.6	(2.7)	16.4	(10.9)	25.4	(20.3)	39.4	(32.1)	101.4	(88.7)	654.4	(975.1)	0.016	(<0.001)
	Men	94.9	134	18.4	(16.7)	ND	(ND)	ND	(ND)	14.2	(10.0)	24.4	(22.1)	34.9	(32.7)	88.3	(87.1)	434.2	(529.7)	0.010	(0.759)
	Women	96.9	156	26.3	(17.7)	ND	(ND)	7.9	(3.7)	17.2	(11.5)	28.3	(19.6)	47.2	(30.5)	112.3	(98.0)	654.4	(975.1)		
	***Minors***	96.9	97	28.3	(28.9)	ND	(ND)	8.1	(5.5)	19.0	(19.0)	30.8	(34.6)	56.2	(56.0)	109.1	(123.7)	150.6	(339.0)		
	Boys	98.1	54	32.1	(38.2)	ND	(ND)	7.9	(9.5)	19.4	(23.5)	32.4	(37.9)	62.9	(71.6)	118.2	(159.0)	150.6	(339.0)	0.257	(0.001)
	Girls	95.5	43	24.2	(20.4)	ND	(ND)	2.0	(1.3)	16.9	(15.4)	30.6	(26.1)	47.2	(37.8)	94.6	(82.1)	135.3	(166.4)		
MCMHP	***Adults***	64.9	290	2.0	(1.6)	ND	(ND)	ND	(ND)	ND	(ND)	4.1	(3.2)	7.7	(6.6)	16.1	(14.6)	167.0	(203.7)	0.067	(0.001)
	Men	66.2	134	2.0	(1.8)	ND	(ND)	ND	(ND)	ND	(ND)	3.9	(3.8)	7.4	(8.0)	14.6	(15.6)	167.0	(203.7)	0.598	(0.085)
	Women	63.8	156	2.1	(1.4)	ND	(ND)	ND	(ND)	ND	(ND)	4.2	(2.9)	8.0	(5.6)	20.5	(13.5)	115.1	(171.4)		
	***Minors***	75.5	97	2.9	(2.9)	ND	(ND)	ND	(ND)	0.9	(1.2)	4.6	(5.6)	10.9	(10.1)	18.7	(24.5)	37.5	(46.2)		
	Boys	81.5	54	3.4	(4.1)	ND	(ND)	ND	(ND)	2.0	(3.1)	6.0	(6.3)	11.7	(11.8)	18.6	(31.9)	24.0	(41.1)	0.244	(0.015)
	Girls	68.2	43	2.3	(1.9)	ND	(ND)	ND	(ND)	ND	(ND)	4.4	(3.9)	9.9	(7.8)	28.8	(22.8)	37.5	(46.2)		
MnBP	***Adults***	87.2	290	12.8	(9.9)	ND	(ND)	ND	(ND)	8.8	(6.0)	18.1	(15.8)	31.4	(28.9)	142.6	(87.4)	5088.2	(6105.8)	0.021	(<0.001)
	Men	87.5	134	10.6	(9.6)	ND	(ND)	ND	(ND)	7.1	(5.5)	13.8	(14.6)	25.5	(28.2)	135.6	(83.9)	5088.2	(6105.8)	0.001	(0.417)
	Women	86.9	156	15.1	(10.2)	ND	(ND)	ND	(ND)	10.7	(7.3)	22.1	(16.9)	40.1	(29.9)	152.9	(118.4)	1780.2	(836.7)		
	***Minors***	92.9	97	17.6	(18.0)	ND	(ND)	ND	(ND)	11.2	(13.8)	23.6	(21.7)	40.6	(43.5)	109.2	(125.8)	194.0	(158.2)		
	Boys	88.9	54	16.4	(19.5)	ND	(ND)	ND	(ND)	10.9	(15.1)	25.1	(29.4)	53.6	(61.3)	146.2	(139.6)	194.0	(158.2)	0.416	(0.016)
	Girls	97.7	43	19.3	(16.2)	ND	(ND)	5.7	(3.0)	13.3	(12.9)	21.0	(16.0)	33.1	(32.0)	76.8	(58.5)	108.0	(78.6)		
MiBP	***Adults***	70.9	290	4.4	(3.4)	ND	(ND)	ND	(ND)	ND	(ND)	9.4	(7.0)	19.1	(17.2)	68.4	(47.4)	129.7	(286.6)	0.032	(<0.001)
	Men	70.6	134	3.7	(3.4)	ND	(ND)	ND	(ND)	ND	(ND)	7.4	(6.6)	17.9	(16.6)	54.5	(59.4)	129.7	(286.6)	0.069	(0.811)
	Women	71.3	156	5.1	(3.4)	ND	(ND)	ND	(ND)	ND	(ND)	11.2	(7.5)	23.2	(17.9)	93.1	(38.8)	115.0	(141.7)		
	***Minors***	80.6	97	7.2	(7.3)	ND	(ND)	ND	(ND)	4.7	(4.9)	13.6	(15.3)	24.2	(29.3)	76.4	(75.0)	138.0	(200.1)		
	Boys	83.3	54	7.8	(9.3)	ND	(ND)	ND	(ND)	6.1	(6.6)	15.0	(18.2)	23.6	(29.7)	72.7	(78.3)	138.0	(200.1)	0.632	(0.109)
	Girls	77.3	43	6.4	(5.4)	ND	(ND)	ND	(ND)	3.0	(2.1)	10.4	(10.3)	27.7	(29.2)	80.7	(79.3)	100.0	(83.3)		
MEP	***Adults***	91.2	290	14.0	(10.8)	ND	(ND)	ND	(ND)	6.7	(5.1)	14.5	(12.2)	33.7	(29.2)	173.8	(199.2)	4675.1	(3286.0)	0.267	(0.479)
	Men	90.4	134	9.9	(9.0)	ND	(ND)	ND	(ND)	4.6	(4.0)	10.4	(9.6)	27.4	(27.9)	145.9	(196.6)	2376.0	(3286.0)	<0.001	(0.055)
	Women	91.9	156	18.7	(12.6)	ND	(ND)	ND	(ND)	9.4	(6.0)	19.6	(13.9)	44.2	(30.0)	204.3	(210.2)	4675.1	(2571.3)		
	***Minors***	92.9	97	11.5	(11.7)	ND	(ND)	ND	(ND)	6.5	(5.5)	12.4	(14.2)	25.3	(29.6)	80.8	(151.3)	412.0	(626.4)		
	Boys	96.3	54	12.9	(15.3)	ND	(ND)	1.3	(1.8)	8.2	(9.3)	13.0	(17.9)	24.8	(33.2)	72.8	(155.9)	248.6	(392.3)	0.468	(0.063)
	Girls	88.6	43	10.0	(8.4)	ND	(ND)	ND	(ND)	5.0	(3.5)	11.1	(9.7)	26.9	(27.0)	222.3	(204.5)	412.0	(626.4)		
MiNP	***Adults***	12.8	290	ND	(ND)	ND	(ND)	ND	(ND)	ND	(ND)	ND	(ND)	ND	(ND)	4.0	(3.5)	39.7	(15.0)	0.005	(0.776)
	Men	9.6	134	ND	(ND)	ND	(ND)	ND	(ND)	ND	(ND)	ND	(ND)	ND	(ND)	3.2	(3.5)	12.1	(15.0)	<0.001	(0.184)
	Women	15.6	156	ND	(ND)	ND	(ND)	ND	(ND)	ND	(ND)	ND	(ND)	ND	(ND)	5.4	(3.6)	39.7	(11.1)		
	***Minors***	12.2	97	ND	(ND)	ND	(ND)	ND	(ND)	ND	(ND)	ND	(ND)	ND	(ND)	9.1	(8.1)	43.4	(49.5)		
	Boys	13.0	54	0.2	(0.3)	ND	(ND)	ND	(ND)	ND	(ND)	ND	(ND)	ND	(ND)	10.2	(12.1)	43.4	(49.5)	0.045	(0.584)
	Girls	11.4	43	ND	(ND)	ND	(ND)	ND	(ND)	ND	(ND)	ND	(ND)	ND	(ND)	13.2	(9.9)	16.9	(22.2)		
MBzP	***Adults***	22.3	290	0.4	(0.3)	ND	(ND)	ND	(ND)	ND	(ND)	ND	(ND)	ND	(ND)	7.8	(5.6)	75.9	(15.7)	0.403	(0.144)
	Men	22.1	134	0.3	(0.3)	ND	(ND)	ND	(ND)	ND	(ND)	ND	(ND)	ND	(ND)	5.4	(5.9)	16.7	(12.8)	0.011	(0.837)
	Women	22.5	156	0.4	(0.3)	ND	(ND)	ND	(ND)	ND	(ND)	ND	(ND)	ND	(ND)	8.4	(5.2)	75.9	(15.7)		
	***Minors***	28.6	97	0.4	(0.4)	ND	(ND)	ND	(ND)	ND	(ND)	ND	(ND)	1.7	(2.1)	7.5	(12.4)	12.7	(27.1)		
	Boys	38.9	54	0.5	(0.6)	ND	(ND)	ND	(ND)	ND	(ND)	ND	(ND)	3.0	(3.0)	11.1	(15.2)	12.7	(27.1)	0.547	(0.007)
	Girls	15.9	43	ND	(ND)	ND	(ND)	ND	(ND)	ND	(ND)	ND	(ND)	0.3	(0.2)	6.4	(5.2)	10.2	(18.3)		
MMP	***Adults***	96.6	290	31.3	(24.2)	ND	(ND)	4.6	(3.8)	15.5	(10.8)	30.9	(23.9)	66.5	(54.3)	308.4	(282.8)	6529.6	(7117.3)	0.094	(0.001)
	Men	97.1	134	29.4	(26.7)	ND	(ND)	3.9	(3.4)	14.7	(12.7)	27.4	(26.1)	59.3	(52.8)	376.5	(324.6)	993.9	(1350.3)	0.339	(0.182)
	Women	96.3	156	33.0	(22.2)	ND	(ND)	6.1	(3.6)	15.9	(9.7)	32.6	(22.3)	70.5	(57.3)	285.7	(258.9)	6529.6	(7117.3)		
	***Minors***	98.0	97	37.5	(38.2)	ND	(ND)	5.1	(4.8)	21.2	(18.1)	41.2	(43.0)	79.0	(83.3)	215.7	(381.8)	1355.1	(1004.9)		
	Boys	100	54	37.4	(44.5)	4.5	(4.5)	5.2	(8.2)	20.9	(22.9)	41.6	(45.9)	70.4	(83.7)	138.5	(392.7)	159.0	(456.4)	0.965	(0.345)
	Girls	95.5	43	37.5	(31.5)	ND	(ND)	1.3	(0.7)	21.2	(11.5)	39.3	(40.0)	81.1	(82.3)	1088.8	(667.6)	1355.1	(1004.9)		

^a^ Abbreviations: MBzP: mono-benzyl phthalate; MiBP: mono-iso-butyl phthalate; MnBP: mono-*n*-butyl phthalate, MCMHP: mono-(2-carboxymethylhexyl) phthalate, MEP: mono-ethyl phthalate, MECPP: mono-(2-ethyl-5-carboxypentyl) phthalate; MEHP: mono-ethylhexyl phthalate; MEHHP: mono-(2-ethyl-5-hydroxyhexyl) phthalate; MEOHP: mono-(2-ethyl-5-oxo-hexyl) phthalate; MMP: mono-methyl phthalate; MiNP: mono-iso-nonyl phthalate; unadjusted level of each phthalate metabolite was shown in the parentheses; ND: not detectable; Detectable rate = number of urine sample with level of each phthalate metabolite above detection limit/ all analyzed urine samples.

^b^ Mann-Whitney U test; p-value for unadjusted level of each phthalate metabolite was shown in the parentheses.

**Fig 3:**
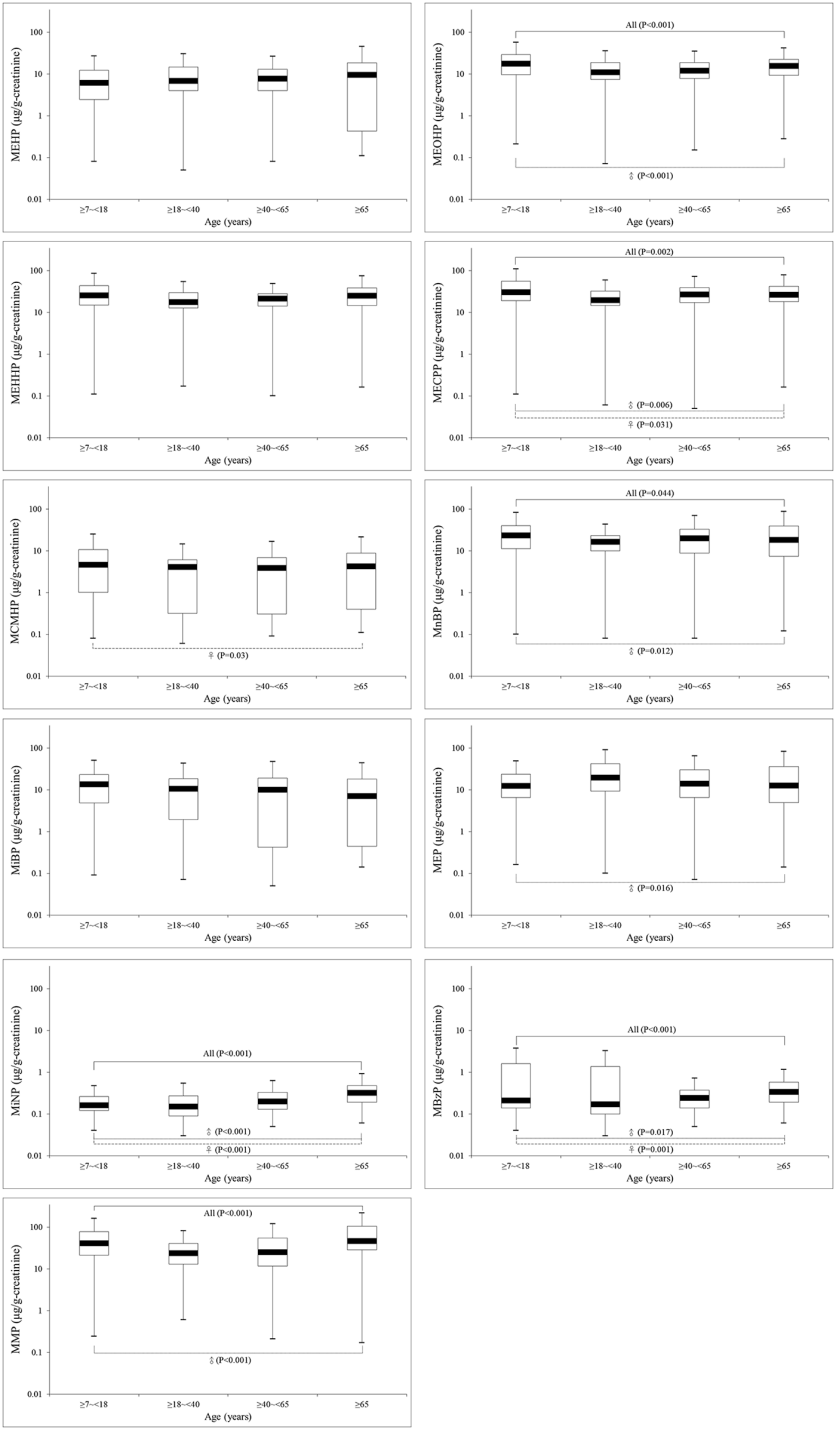
Distribution of 11 phthalate metabolites in a sample of the general Taiwanese population (N = 387) by age groups (solid line inside the box plot: median).

Fig 3 showed the distribution and median levels of phthalate metabolites in our sample of the general Taiwanese population classified into four age groups. We found that J-shape curves of the exposure levels of MEOHP (P<0.001), MECPP(P = 0.002), MnBP (P = 0.044), MiNP (P<0.001), MBzP (P<0.001) and MMP (P<0.001) in our subjects were significantly varied by age regardless of gender, which indicated minors may expose to higher level of these phthalates than adults. The median level of MEP (creatinine-unadjusted) at age 18–40 years was significantly (P<0.001) higher than the other age groups, especially for women. Besides, median levels of MnBP and MiBP in all subjects decreased along with increasing age. No significant change of MEHP in our subjects was observed regardless of age or gender. Detailed data were shown in the S2 Table.

We further categorized our subjects according to five regions (S3 Table). We found that the median levels of MEHP, MEHHP, MEOHP, MECPP, MCMHP, MiBP, MEP, MBzP, and MMP were statistically significant (p<0.001) varied by region. Except for the remote island region, we observed that the median levels of MEHP, MECPP, MCMHP, MnBP, MiBP and MBzP in southern Taiwan were highest among four regions. We also observed a 2-3-fold higher median level of MMP in the remote island was higher than other regions. The median levels of MiNP and MBzP in all areas were below the detection limit.

### Comparison of phthalate exposure among different countries

**Fig 4:**
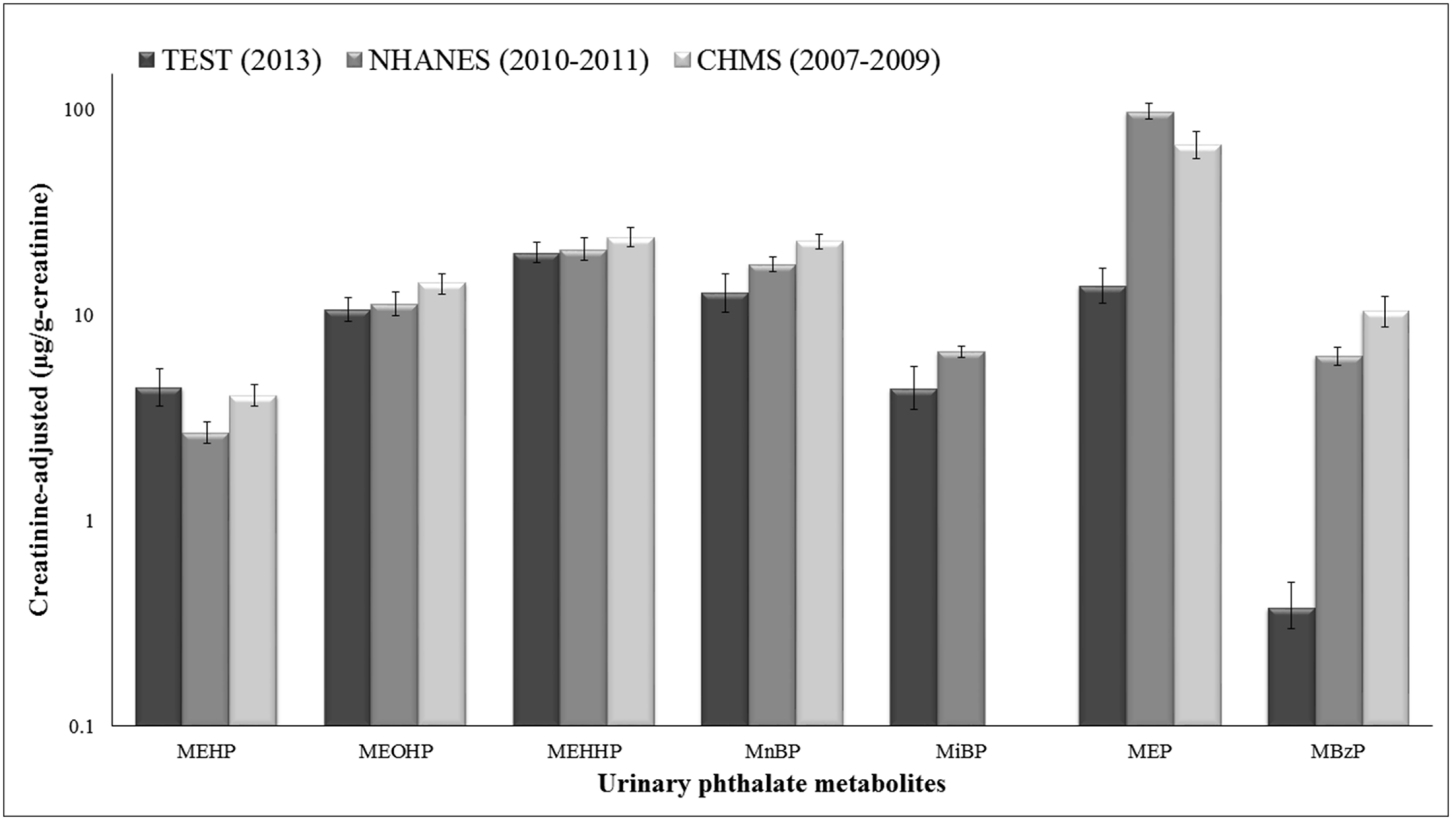
Comparison of urinary levels of seven phthalate metabolites in a general population sample of Taiwanese (≧7 years), Americans (>6 years) and Canadians (18–49 years).

Fig 4 showed a comparison of phthalate metabolite levels in the general population between Taiwan, Canada [20], and the United States [27]. We found that the geometric mean (GM) level of MEHP in the Taiwanese population sample was 1.6-fold higher than those in the NHANES 10–11. The GM levels of secondary MEHP metabolites and MnBP in this study were comparable to those reported in the CHMS 07–08 and NHANES 10–11. The exposure levels of MEP and MBzP were approximately 5–7 times lower than those in CHMS 07–08 and NHANES 10–11.

## Discussion

This is the first study systematically evaluating phthalate exposure in a sample of the general Taiwanese population. We found a significant difference in phthalate exposure according to age and gender. Minors (≧7~<18 years old) and elders (≧ 65 years old) tended to have exposure to higher levels of most phthalates, such as DEHP, DnBP, BBzP and DMP. Women (≧18 years old) were exposed to higher levels of DEHP, DBP (in particular DnBP) and DEP than men (≧18 years old) in Taiwan. Minors had significantly higher urinary levels of MEHHP, MEOHP, MECPP, MnBP and MiBP than the adult population, especially for boys.

High detectable rates (>70%) of MEHP, MEOHP, MEHHP, MECPP, MnBP, MiBP, MEP and MMP were observed in this sample of the general Taiwanese population. Due to the short-half-lives of most phthalates, DEHP, DnBP, DiBP, DEP, and DMP are frequently present in the daily life of the general Taiwanese population. Over 73% of participating adults and minors frequently used food preservation film and plastic bag for food storage (S4 Table). About 73% and 43% of minors and adults, respectively, used body wash every day, whereas less than 2% of all participants frequently used perfume and nail polish; these results were not consistent with other studies [28–29]. Around 42% and 13% of minors and adults, respectively, took at least one medicine constantly during the past one month. About 25% of participants reported using pesticides at home. Plastic products related to food preservation, body wash, medicine, and pesticide usage were potential sources of different phthalates exposure in the general Taiwanese population.

A low detection rate of MiNP (11.1%) and MBzP (22.4%) in Taiwanese was observed regardless of different age groups and gender. Although DINP is a substitute for DEHP in plastics, DINP is not widely used for plastics related to products used daily in Taiwan or other Asian countries. Besides, MiNP is not the major DINP metabolite as it is converted into secondary metabolites [30]. Thus, the low MiNP detection rate does not imply low DINP exposure in the general Taiwanese population. It is also important to note that new plastic phthalate substitutes have been developed; their use may increase rapidly in the near future [31]. Monitoring secondary metabolites of DINP is worthy of further investigation in studies of Asian countries. BBzP is primarily used in PVC-based floor materials, which are not used as much for Taiwanese home decoration as in previous years. Due to MBzP being a major metabolite of BBzP, low detection of MBzP suggests low-exposure levels of BBzP in the general Taiwanese population.

We analyzed the difference of creatinine levels in our subjects by age and gender (S5 Table). We found that male subjects had a significantly higher median level of creatinine than females (102.4 vs. 71 mg/dl, p<0.001) regardless of age difference. We further excluded 24 subjects with creatinine<30 or ≧300 mg/dl for further analysis, and the results were consistent. Our study revealed that we may partially diminish the difference of phthalate exposure between males and females when using a creatinine-adjustment-based method for comparison. However, we found a significantly high correlation (R = 0.83~0.96, P<0.001, S6 Table) between creatinine-adjusted and unadjusted concentrations of each phthalate metabolites using Pearson correlation analysis. Hence, in order to adjust for individual urinary excretion rate, we primarily presented our data in a creatinine-based level.

We found that urinary levels of DEHP metabolites in minor Taiwanese boys were significantly higher than those of most adult age groups. For minors, the behaviors of hand-to-mouth exposure and use of drinking containers are the likely causes of major phthalate exposure [32]. DEHP is of great concern in boys due to its antiandrogenic effects [33]; hence, intervention strategies for reducing phthalate exposure in boys warrant further investigation. Besides, we observed that urinary MECPP and ΣDEHP metabolites levels in elderly Taiwanese women (≧65 years) were higher than in any other age group. Taiwanese elderly women use food preservation films to save leftover food heated in a microwave oven; some food preservation films are made with PVC, which may increase DEHP concentration in meals [34]. Another possibility is that increased phthalate levels may correlate with increased amounts of cosmetic, perfume, and personal care product usage [28–29] in women. Only 45% of female adults frequently used lotion and body wash every day, whereas less than 5% of them used perfume and nail polish frequently (S4 Table). We suggested that food preservation and personal care products are possible sources of DEHP exposure in elderly Taiwanese women.

One study reported that GM levels of urinary MEHP, MEHHP, MEOHP, MEP, MBP were around 9.87, 50.9, 24.1, 19.8 and 55.9 μg/g creatinine, respectively, in adult Taiwanese during 2005–08 [35], two times higher than those in the current study. However, no information was available about potential phthalate exposure sources in the Taiwanese study from 2005–08. Another study also reported median level of urinary MEHP was 29.8 μg/g creatinine in 225 school children of Taipei city during 2009–10, four times higher than those in the current study [36]. Our sample of the general Taiwanese population provided some evidence of decreasing DEHP and DBP levels after enacted restrictions and regulations. In adult Taiwanese women, the median levels of most phthalate metabolites in age groups between 18 to 65 years were higher than those in men. For reproductive age women (18–40 years), some studies reported DEHP and DBP exposure in pregnant women may alter human hormones [37–38], fetal and child development [39–40], and pregnancy losses [41], endometriosis [42] or leiomyoma [43–44]. Although limited evidence showed that exposure levels of most phthalate metabolites significantly decreased after enacted restrictions and regulation [10], DEHP exposure levels in reproductive-aged Taiwanese women remain higher than those reported from other countries, warranting further investigation.

We found that both children and adolescents are exposed to higher levels of phthalates, a finding consistent with other studies [20, 23, 27, 43–44]. The Canadian study reported children had significantly higher urinary concentrations of metabolites of DEHP, DnBP and BBzP than adolescents and adults [20]; similar results were also observed in German and NHANES studies [27,44]. Increasing numbers of studies have reported endocrine, immunologic, and neurological effects of both prenatal and postnatal phthalates exposure in young children [11–12, 16–17, 40, 45]. Hence, regulating human biomonitoring value and intervention strategies of consumer behavior [32, 46] were necessary to reduce phthalate exposure in children.

Some studies revealed good correlations with primary and secondary DEHP metabolites in US and German populations [30, 44]. In adults, we observed a significantly low correlation between MEHP and three secondary DEHP metabolites (S6 Table), whereas we found moderate correlation between MEHHP and MECPP (R = 0.562, *P*<0.001) as well as MEOHP and MCMHP (R = 0.509, *P*<0.001). Our data showed a high correlation between MEHHP and ΣDEHP metabolites (R = 0.802, *P*<0.001), which indicated a good surrogate exposure index of DEHP in the Taiwanese population. In minors, we found a high correlation among MEOHP (R = 0.802, *P*<0.001), MEHHP (R = 0.782, *P*<0.001), MECPP, and ΣDEHP metabolites (S6 Table). Although metabolic abilities may differentiate between age groups, we suggested MEHHP could be a good biomarker of DEHP in the general Taiwanese population.

Our current study demonstrates several strengths. First, our study was a nationwide sample of subjects, including children ≥7 years old. Second, we also collected relevant factors from our participants through interview questionnaires, allowing further analysis of potential sources of phthalate exposure. Third, we determined the internal exposure in the general population which supplied a basis for estimating phthalate-related health risks. Our data provided reference values of phthalates in the Taiwanese population.

Some limitations of our study should be noticed in relation to data interpretation. First, this is a cross-sectional study with one-time measurements. The distribution of individual phthalate exposure can vary, and therefore further study is needed for temporal assessment over time. Second, we did not have sufficient evidence to link decreased phthalate exposure to this episodes. But, enacted restrictions and regulations on phthalates can possibly decrease phthalate exposure levels from many products in the general population.

We concluded that exposure levels of phthalates varied by age and gender, and were possibly affected by different lifestyles. Enacted restrictions and regulations could effectively reduce phthalate exposure in the general population; however, continuous biomonitoring surveillance is necessary to detect the long-term level variation. Although the DEHP exposure profile dramatically dropped after strict regulation, young boys and women in Taiwan were still exposed to higher levels of certain phthalates. These related health influences, such as reproductive development, are worthy of further investigation.

## Supporting Information

S1 Table Main phthalate products and urinary metabolites investigated in this study. (DOCX)

S2 Table Detectable rate and distribution of creatinine-adjusted levels (μg/g creatinine, or ng/ml) of phthalate metabolites ^a^ in a sample of the general Taiwanese population (N = 387) by age groups. (DOCX)

S3 Table Distribution of creatinine-adjusted levels (μg/g creatinine or ng/ml) in phthalate metabolites ^a^ from a sample of the general Taiwanese population by different regions (N = 387). (DOCX)

S4 Table Number ^a^ of individuals who use phthalate-containing food-related plastic products, cosmetic and personal care products, medicine and pesticides. (DOCX)

S5 Table Differences in subject creatinine levels (mg/dl) by age and gender (N = 387) in our study. (DOCX)

S6 Table Pearson correlation coefficients among 11 urinary phthalate metabolites (creatinine-adjusted) in Taiwanese minors (n = 97, upper right) and Taiwanese adults (n = 290, age≧18 years old, lower left), and adjusted/unadjusted creatinine levels (diagonal) for all subjects. (DOCX)

## References

[1] Schettler T. Human exposure to phthalates via consumer products. Int J Androl 2006;29:134–9. 16466533 10.1111/j.1365-2605.2005.00567.x

[2] Zota AR, Calafat AM, Woodruff TJ. Temporal trends in phthalate exposures: findings from the National Health and Nutrition Examination Survey, 2001–2010. Environ Health Perspect. 2014;122: 235–41. 10.1289/ehp.1306681 24425099 PMC3948032

[3] Wu MT, Wu CF, Wu JR, Chen BH, Chen EK, Chao MC, et al The public health threat of phthalate-tainted foodstuffs in Taiwan: the policies the government implemented and the lessons we learned. Environ Int. 2012;44:75–9. 10.1016/j.envint.2012.01.014 22361240

[4] Li JH, Ko YC. Plasticizer incident and its health effects in Taiwan. Kaohsiung J Med Sci. 2012; 28(7 Suppl):S17–21. 10.1016/j.kjms.2012.05.005 22871596 PMC11922130

[5] Huang PC, Liou SH, Ho IK, Chiang HC, Huang HI, Wang SL. Phthalates Exposure and Endocrinal Effects: An Epidemiological Review. J Food Drug Anal. 2012; 20: 719–33.

[6] Jurewicz J, Hanke W. Exposure to phthalates: reproductive outcome and children health. A review of epidemiological studies. Int J Occup Med Environ Health. 2011; 24: 115–41. 10.2478/s13382-011-0022-2 21594692

[7] Meeker JD, Ferguson KK. Relationship between urinary phthalate and bisphenol A concentrations and serum thyroid measures in U.S. adults and adolescents from the National Health and Nutrition Examination Survey (NHANES) 2007–2008. Environ Health Perspect. 2011;119:1396–402. 10.1289/ehp.1103582 21749963 PMC3230451

[8] Huang PC, Kuo PL, Chou YY, Lin SJ, Lee CC. Associations between Urinary Phthalate Monoesters and Thyroid Hormones in Pregnant Women. Hum Reprod. 2007; 22: 2715–22. 17704099 10.1093/humrep/dem205

[9] Boas M, Frederiksen H, Feldt-Rasmussen U, Skakkebæk NE, Hegedüs L, Hilsted L, et al Childhood exposure to phthalates: associations with thyroid function, insulin-like growth factor I, and growth. Environ Health Perspect. 2010;118:1458–64. 10.1289/ehp.0901331 20621847 PMC2957929

[10] Wu MT, Wu CF, Chen BH, Chen EK, Chen YL, Shiea J, et al Intake of phthalate-tainted foods alters thyroid functions in Taiwanese children. PLoS One. 2013; 8: e55005 10.1371/journal.pone.0055005 23383031 PMC3559382

[11] Ku HY, Su PH, Wen HJ, Sun HL, Wang CJ, Chen HY, et al Prenatal and postnatal exposure to phthalate esters and asthma: a 9-year follow-up study of a Taiwanese birth cohort. PLoS One. 2015; 10: e0123309 10.1371/journal.pone.0123309 25875379 PMC4395154

[12] Wang IJ, Lin CC, Lin YJ, Hsieh WS, Chen PC. Early life phthalate exposure and atopic disorders in children: a prospective birth cohort study. Environ Int. 2014; 62: 48–54. 10.1016/j.envint.2013.09.002 24161446

[13] Huang PC, Tsai EM, Li WF, Liao PC, Chung MC, Wang YH, et al Association between Phthalate Exposure and Glutathione S-transferase (GST) M1 Polymorphism in Adenomyosis, Leiomyoma, and Endometriosis. Hum Reprod. 2010; 25: 986–94. 10.1093/humrep/deq015 20147336

[14] Mendiola J, Meeker JD, Jørgensen N, Andersson AM, Liu F, Calafat AM, et al Urinary concentrations of di(2-ethylhexyl) phthalate metabolites and serum reproductive hormones: pooled analysis of fertile and infertile men. J Androl. 2012; 33: 488–98. 10.2164/jandrol.111.013557 21597090 PMC3433231

[15] Huang PC, Li WF, Liao PC, Sun CW, Tsai EM, Wang SL. Risk for estrogen-dependent diseases in relation to phthalate exposure and polymorphisms of CYP17A1 and estrogen receptor genes. Environ Sci Pollut Res Int. 2014; 21: 13964–73. 10.1007/s11356-014-3260-6 25030786

[16] Zhang Y, Meng X, Chen L, Li D, Zhao L, Zhao Y, et al Age and sex-specific relationships between phthalate exposures and obesity in Chinese children at puberty. PLoS One. 2014; 9: e104852 10.1371/journal.pone.0104852 25121758 PMC4133266

[17] Smerieri A, Testa C, Lazzeroni P, Nuti F, Grossi E, Cesari S, et al Di-(2-ethylhexyl) phthalate metabolites in urine show age-related changes and associations with adiposity and parameters of insulin sensitivity in childhood. PLoS One. 2015; 10: e0117831 10.1371/journal.pone.0117831 25706863 PMC4338209

[18] Bai PY, Wittert GA, Taylor AW, Martin SA, Milne RW, Shi Z. The association of socio-demographic status, lifestyle factors and dietary patterns with total urinary phthalates in Australian men. PLoS One. 2015; 10: e0122140 10.1371/journal.pone.0122140 25875472 PMC4398403

[19] Becker K, Seiwert M, Casteleyn L, Joas R, Joas A, Biot P, et al A systematic approach for designing a HBM pilot study for Europe. Int J Hyg Environ Health. 2014; 217: 312–22. 10.1016/j.ijheh.2013.07.004 23928002

[20] Saravanabhavan G, Guay M, Langlois É, Giroux S, Murray J, Haines D. Biomonitoring of phthalate metabolites in the Canadian population through the Canadian Health Measures Survey (2007–2009). Int J Hyg Environ Health. 2013; 216: 652–61. 10.1016/j.ijheh.2012.12.009 23419587

[21] Ha M, Kwon HJ, Leem JH, Kim HC, Lee KJ, Park I, et al Korean Environmental Health Survey in Children and Adolescents (KorEHS-C): survey design and pilot study results on selected exposure biomarkers. Int J Hyg Environ Health. 2014; 217: 260–70. 10.1016/j.ijheh.2013.06.001 23831304

[22] Schindler BK, Esteban M, Koch HM, Castano A, Koslitz S, Cañas A, et al The European COPHES/DEMOCOPHES project: towards transnational comparability and reliability of human biomonitoring results. Int J Hyg Environ Health. 2014; 217: 653–61. 10.1016/j.ijheh.2013.12.002 24405937

[23] Dewalque L, Pirard C, Charlier C. Measurement of urinary biomarkers of parabens, benzophenone-3, and phthalates in a Belgian population. Biomed Res Int. 2014; 2014: 649314 10.1155/2014/649314 24719881 PMC3955696

[24] Frederiksen H, Jensen TK, Jørgensen N, Kyhl HB, Husby S, Skakkebæk NE, et al Human urinary excretion of non-persistent environmental chemicals: an overview of Danish data collected between 2006 and 2012. Reproduction. 2014; 147: 555–65. 10.1530/REP-13-0522 24395915

[25] Pan WH, Wu HJ, Yeh CJ, Chuang SY, Chang HY, Yeh NH, et al Diet and health trends in Taiwan: comparison of two nutrition and health surveys from 1993–1996 and 2005–2008. Asia Pac J Clin Nutr. 2011; 20: 238–50. 21669593

[26] Koch HM, Gonzalez-Reche LM, Angerer J. On-line clean-up by multidimensional liquid chromatography-electrospray ionization tandem mass spectrometry for high throughput quantification of primary and secondary phthalate metabolites in human urine. J Chromatogr B Analyt Technol Biomed Life Sci. 2003; 784: 169–82. 12504195 10.1016/s1570-0232(02)00785-7

[27] CDC 2012. Fourth national report on human exposure to environmental chemicals, updated tables. Available: http://www.cdc.gov/exposurereport/pdf/FourthReport_UpdatedTables _Feb2015.pdf. Accessed 2015 Feb 1.

[28] Parlett LE, Calafat AM, Swan SH. Women's exposure to phthalates in relation to use of personal care products. J Expo Sci Environ Epidemiol. 2013; 23:197–206. 10.1038/jes.2012.105 23168567 PMC4097177

[29] Philippat C, Bennett D, Calafat AM, Picciotto IH. Exposure to select phthalates and phenols through use of personal care products among Californian adults and their children. Environ Res. 2015; 140: 369–76. 10.1016/j.envres.2015.04.009 25929801 PMC4724203

[30] Calafat AM, Wong LY, Silva MJ, Samandar E, Preau JL Jr, Jia LT, et al Selecting adequate exposure biomarkers of diisononyl and diisodecyl phthalates: data from the 2005–2006 National Health and Nutrition Examination Survey. Environ Health Perspect. 2011; 119: 50–5. 10.1289/ehp.1002316 20870567 PMC3018499

[31] Schütze A, Kolossa-Gehring M, Apel P, Brüning T, Koch HM. Entering markets and bodies: increasing levels of the novel plasticizer Hexamoll DINCH in 24 h urine samples from the German Environmental Specimen Bank. Int J Hyg Environ Health. 2014; 217: 421–6. 10.1016/j.ijheh.2013.08.004 24029725

[32] Chen CY, Chou YY, Lin SJ, Lee CC. Developing an intervention strategy to reduce phthalate exposure in Taiwanese girls. Sci Total Environ. 2015; 517: 125–131. 10.1016/j.scitotenv.2015.02.021 25725197

[33] Swan SH. Environmental phthalate exposure in relation to reproductive outcomes and other health endpoints in humans. Environ Res. 2008; 108: 177–84. 18949837 10.1016/j.envres.2008.08.007 PMC2775531

[34] Chen ML, Chen JS, Tang CL, Mao IF. The internal exposure of Taiwanese to phthalate—an evidence of intensive use of plastic materials. Environ Int. 2008; 34:79–85. 17765308 10.1016/j.envint.2007.07.004

[35] Huang PC, Liou SH, Sun CW, Pan WH, Wang CJ, Wang SL. Human Biomonitoring for Phthalates Exposure from Nutrition and Health Survey in Taiwan (NAHSIT). International Society of Exposure Science conference, proceeding, Seattle, WA, USA; 2012.

[36] Bao J, Zeng XW, Qin XD, Lee YL, Chen X, Jin YH, et al Phthalate Metabolites in Urine Samples from School Children in Taipei, Taiwan. Arch Environ Contam Toxicol. 2015 3 8 10.1007/s00244-015-0146-7 25749906

[37] Lin LC, Wang SL, Chang YC, Huang PC, Cheng JT, Su PH, et al Associations between maternal phthalate exposure and cord sex hormones in human infants. Chemosphere. 2011; 83: 1192–9. 10.1016/j.chemosphere.2010.12.079 21272909

[38] Sathyanarayana S, Barrett E, Butts S, Wang C, Swan SH. Phthalate exposure and reproductive hormone concentrations in pregnancy. Reproduction. 2014; 147: 401–9. 10.1530/REP-13-0415 24196015 PMC3943643

[39] Swan SH, Sathyanarayana S, Barrett ES, Janssen S, Liu F, Nguyen RH, et al First trimester phthalate exposure and anogenital distance in newborns. Hum Reprod. 2015; 30: 963–72. 10.1093/humrep/deu363 25697839 PMC4359397

[40] Kobrosly RW, Evans S, Miodovnik A, Barrett ES, Thurston SW, Calafat AM, et al Prenatal phthalate exposures and neurobehavioral development scores in boys and girls at 6–10 years of age. Environ Health Perspect. 2014; 122: 521–8. 10.1289/ehp.1307063 24577876 PMC4014764

[41] Toft G, Jönsson BA, Lindh CH, Jensen TK, Hjollund NH, Vested A, et al Association between pregnancy loss and urinary phthalate levels around the time of conception. Environ Health Perspect. 2012; 120: 458–63. 10.1289/ehp.1103552 22113848 PMC3295336

[42] Reddy BS, Rozati R, Reddy BV, Raman NV. Association of phthalate esters with endometriosis in Indian women. BJOG. 2006; 113: 515–20. 16637895 10.1111/j.1471-0528.2006.00925.x

[43] Becker K, Göen T, Seiwert M, Conrad A, Pick-Fuss H, Müller J, et al GerES IV: phthalate metabolites and bisphenol A in urine of German children. Int J Hyg Environ Health. 2009; 212: 685–92. 10.1016/j.ijheh.2009.08.002 19729343

[44] Koch HM, Wittassek M, Brüning T, Angerer J, Heudorf U. Exposure to phthalates in 5–6 years old primary school starters in Germany—a human biomonitoring study and a cumulative risk assessment. Int J Hyg Environ Health. 2011; 214: 188–95. 10.1016/j.ijheh.2011.01.009 21371937

[45] Téllez-Rojo MM, Cantoral A, Cantonwine DE, Schnaas L, Peterson K, Hu H, et al Prenatal urinary phthalate metabolites levels and neurodevelopment in children at two and three years of age. Sci Total Environ. 2013; 461–462: 386–90. 10.1016/j.scitotenv.2013.05.021 23747553 PMC3735862

[46] Schulz C, Wilhelm M, Heudorf U, Kolossa-Gehring M, Human Biomonitoring Commission of the German Federal Environment Agency. Update of the reference and HBM values derived by the German Human Biomonitoring Commission. Int J Hyg Environ Health. 2011; 215: 26–35. 21820957 10.1016/j.ijheh.2011.06.007

