# Supplementary material for: Age and Gender Differences in Urinary Levels of Eleven Phthalate Metabolites in General Taiwanese Population after a DEHP Episode
Source: PLoS One. 2015 Jul 24;10(7):e0133782. doi: 10.1371/journal.pone.0133782 (PMC4514596; doi:10.1371/journal.pone.0133782)
Supplement: S1 Table — (DOCX) [file pone.0133782.s001.docx]

**S1 Table. Main phthalate products and urinary metabolites investigated in this study.**

| Phthalates | Urinary Metabolites | Main Application |
| --- | --- | --- |
| Di-2-ethylhexyl phthalate (DEHP) | Mono-ethylhexyl phthalate (MEHP), Mono-(2-ethyl-5-oxo-hexyl) phthalate (MEOHP), Mono-(2-ethyl-5-hydroxyhexyl) phthalate (MEHHP), Mono-(2-ethyl-5-carboxypentyl) phthalate (MECPP), Mono-(2-carboxymethylhexyl) phthalate (MCMHP) | Plastic products, cables, children's toys, medical blood bags |
| Di-n-butyl phthalate (DnBP) | Mono-*n*-butyl phthalate (MnBP) | Food package film, plastic products |
| Di-isobutyl phthalate (DiBP) | Mono-iso-butyl phthalate (MiBP) | Plastic products |
| Diethyl phthalate (DEP) | Mono-ethyl phthalate (MEP) | Hair spray, body wash, perfume, lotion, deodorant |
| Di-isononyl phthalate (DiNP) | Mono-iso-nonyl phthalate (MiNP) | Cable, plastics |
| Butyl benzyl phthalate (BBzP) | Mono-benzyl phthalate (MBzP) | PVC floor |
| Dimethyl phthalate (DMP) | Mono-methyl phthalate (MMP) | Insect repellent |
