# Supplementary material for: Age and Gender Differences in Urinary Levels of Eleven Phthalate Metabolites in General Taiwanese Population after a DEHP Episode
Source: PLoS One. 2015 Jul 24;10(7):e0133782. doi: 10.1371/journal.pone.0133782 (PMC4514596; doi:10.1371/journal.pone.0133782)
Supplement: S2 Table — (DOCX) [file pone.0133782.s002.docx]

**S2 Table. Detectable rate and distribution of creatinine-adjusted levels (μg/g creatinine or ng/ml) of phthalate metabolites ^a^ in a sample of the general Taiwanese population (N=387) by age groups.**

| Phthalate metabolites | Group | Detectable rate (%) | *n* | GM | | Min | | 5th | | 25th | | 50th | | 75th | | 95th | | Max | | *P*-value ^b^ | |
| --- | --- | --- | --- | --- | --- | --- | --- | --- | --- | --- | --- | --- | --- | --- | --- | --- | --- | --- | --- | --- | --- |
|  |  |  |  |  |  |  |  |  |  |  |  |  |  |  |  |  |  |  |  |  |  |
| MEHP | **All** | 77.4 | 387 | 4.3 | (3.6) | ND | (ND) | ND | (ND) | 2.4 | (2.4) | 7.4 | (6.7) | 14.7 | (12.2) | 33.5 | (30.2) | 110.3 | (138.9) | 0.322 | (0.066) |
|  | ≥7~<18 | 80.6 | 97 | 4.0 | (4.1) | ND | (ND) | ND | (ND) | 2.4 | (3.1) | 6.1 | (7.2) | 12.5 | (12.2) | 27.2 | (27.5) | 74.3 | (69.1) |  |  |
|  | ≥18~<40 | 80.8 | 73 | 4.1 | (4.7) | ND | (ND) | ND | (ND) | 3.9 | (3.2) | 6.8 | (8.6) | 14.9 | (16.0) | 30.2 | (38.5) | 93.2 | (138.9) |  |  |
|  | ≥40~<65 | 78.2 | 124 | 4.6 | (3.5) | ND | (ND) | ND | (ND) | 4.0 | (2.7) | 7.8 | (6.3) | 13.7 | (11.3) | 31.5 | (27.4) | 110.3 | (78.8) |  |  |
|  | ≥65 | 70.2 | 93 | 4.5 | (2.6) | ND | (ND) | ND | (ND) | ND | (ND) | 9.6 | (5.9) | 18.7 | (10.8) | 45.8 | (30.8) | 105.7 | (62.9) |  |  |
|  | **Males** | 79.5 | 188 | 4.3 | (4.3) | ND | (ND) | ND | (ND) | 2.7 | (3.1) | 7.3 | (7.6) | 14.4 | (14.1) | 31.1 | (37.0) | 74.3 | (69.1) | 0.439 | (0.316) |
|  | ≥7~<18 | 88.9 | 54 | 5.2 | (6.3) | ND | (ND) | ND | (ND) | 3.2 | (4.6) | 6.6 | (8.6) | 13.8 | (15.7) | 27.3 | (36.5) | 74.3 | (69.1) |  |  |
|  | ≥18~<40 | 73.5 | 33 | 3.0 | (4.0) | ND | (ND) | ND | (ND) | ND | (ND) | 5.0 | (8.9) | 14.8 | (17.1) | 30.2 | (44.5) | 31.3 | (55.7) |  |  |
|  | ≥40~<65 | 77.4 | 53 | 4.0 | (3.9) | ND | (ND) | ND | (ND) | 3.0 | (2.8) | 7.3 | (7.1) | 12.5 | (13.9) | 31.9 | (28.2) | 42.9 | (58.5) |  |  |
|  | ≥65 | 75.5 | 48 | 4.8 | (3.2) | ND | (ND) | ND | (ND) | 1.0 | (0.6) | 9.8 | (6.2) | 18.3 | (11.2) | 37.2 | (39.9) | 51.0 | (62.9) |  |  |
|  | **Females** | 75.5 | 199 | 4.3 | (3.0) | ND | (ND) | ND | (ND) | 0.8 | (0.2) | 7.6 | (6.0) | 15.1 | (11.1) | 35.9 | (27.0) | 110.3 | (138.9) | 0.271 | (0.070) |
|  | ≥7~<18 | 70.5 | 43 | 2.8 | (2.4) | ND | (ND) | ND | (ND) | ND | (ND) | 5.3 | (5.9) | 11.2 | (8.7) | 30.0 | (24.8) | 37.2 | (27.1) |  |  |
|  | ≥18~<40 | 86.4 | 40 | 5.2 | (5.5) | ND | (ND) | ND | (ND) | 4.1 | (3.7) | 7.9 | (8.3) | 15.0 | (15.9) | 34.2 | (31.2) | 93.2 | (138.9) |  |  |
|  | ≥40~<65 | 78.9 | 71 | 5.0 | (3.3) | ND | (ND) | ND | (ND) | 4.0 | (2.7) | 8.0 | (5.2) | 15.4 | (10.8) | 31.2 | (27.6) | 110.3 | (78.8) |  |  |
|  | ≥65 | 64.4 | 45 | 4.3 | (2.0) | ND | (ND) | ND | (ND) | ND | (ND) | 9.5 | (5.1) | 20.2 | (9.3) | 67.0 | (23.1) | 105.7 | (31.0) |  |  |
| MEOHP | **All** | 94.7 | 387 | 12.0 | (9.9) | ND | (ND) | ND | (ND) | 8.4 | (6.4) | 13.5 | (11.5) | 21.7 | (20.3) | 50.6 | (52.9) | 244.5 | (289.6) | <0.001 | (<0.001) |
|  | ≥7~<18 | 99 | 97 | 16.9 | (17.2) | ND | (ND) | 4.3 | (4.2) | 9.6 | (9.7) | 17.8 | (19.1) | 29.1 | (31.3) | 61.5 | (80.7) | 140.2 | (150.0) |  |  |
|  | ≥18~<40 | 96.2 | 73 | 10.2 | (11.9) | ND | (ND) | 1.6 | (1.7) | 7.4 | (8.0) | 11.0 | (12.0) | 18.7 | (23.2) | 36.5 | (64.2) | 194.4 | (289.6) |  |  |
|  | ≥40~<65 | 90.3 | 124 | 9.1 | (7.0) | ND | (ND) | ND | (ND) | 7.8 | (5.1) | 12.0 | (9.9) | 18.9 | (16.3) | 30.8 | (29.6) | 56.5 | (55.4) |  |  |
|  | ≥65 | 94.7 | 93 | 13.7 | (7.7) | ND | (ND) | ND | (ND) | 9.4 | (5.3) | 15.6 | (9.1) | 22.4 | (13.6) | 64.2 | (36.9) | 244.5 | (178.8) |  |  |
|  | **Males** | 95.8 | 188 | 12.1 | (11.9) | ND | (ND) | 2.3 | (2.4) | 8.3 | (7.6) | 12.8 | (13.4) | 21.5 | (24.2) | 54.4 | (65.6) | 146.5 | (178.8) | <0.001 | (<0.001) |
|  | ≥7~<18 | 100 | 54 | 19.3 | (23.0) | 3.4 | (3.5) | 5.1 | (5.2) | 11.3 | (15.0) | 19.8 | (24.4) | 36.3 | (33.6) | 68.7 | (108.5) | 140.2 | (150.0) |  |  |
|  | ≥18~<40 | 94.1 | 33 | 7.5 | (9.9) | ND | (ND) | ND | (ND) | 4.9 | (6.7) | 8.5 | (13.1) | 16.2 | (20.8) | 29.3 | (45.6) | 30.2 | (74.2) |  |  |
|  | ≥40~<65 | 92.5 | 53 | 9.5 | (9.1) | ND | (ND) | ND | (ND) | 7.4 | (6.2) | 12.0 | (12.0) | 17.7 | (19.5) | 26.6 | (32.2) | 30.5 | (43.7) |  |  |
|  | ≥65 | 95.9 | 48 | 12.8 | (8.6) | ND | (ND) | 2.4 | (1.1) | 9.3 | (5.3) | 13.7 | (9.6) | 21.2 | (15.0) | 65.9 | (96.9) | 146.5 | (178.8) |  |  |
|  | **Females** | 93.6 | 199 | 11.9 | (8.4) | ND | (ND) | ND | (ND) | 8.4 | (5.8) | 14.5 | (9.9) | 21.8 | (16.3) | 50.2 | (35.1) | 244.5 | (289.6) | 0.068 | (<0.001) |
|  | ≥7~<18 | 97.7 | 43 | 14.2 | (11.9) | ND | (ND) | 4.2 | (2.5) | 8.7 | (6.6) | 16.2 | (15.1) | 25.4 | (21.1) | 54.9 | (59.4) | 72.8 | (68.9) |  |  |
|  | ≥18~<40 | 97.7 | 40 | 13.2 | (13.9) | ND | (ND) | 4.5 | (2.5) | 8.4 | (8.8) | 12.7 | (12.0) | 19.9 | (26.4) | 64.3 | (64.6) | 194.4 | (289.6) |  |  |
|  | ≥40~<65 | 88.7 | 71 | 8.8 | (5.8) | ND | (ND) | ND | (ND) | 8.1 | (4.7) | 11.4 | (8.6) | 19.3 | (12.7) | 35.7 | (25.6) | 56.5 | (55.4) |  |  |
|  | ≥65 | 93.3 | 45 | 14.7 | (6.9) | ND | (ND) | ND | (ND) | 10.5 | (5.4) | 17.5 | (8.3) | 23.7 | (12.9) | 71.1 | (30.5) | 244.5 | (33.6) |  |  |

| Phthalate metabolites | Group | Detectable rate (%) | *n* | GM | | Min | | 5th | | 25th | | 50th | | 75th | | 95th | | Max | | *P*-value ^b^ | |
| --- | --- | --- | --- | --- | --- | --- | --- | --- | --- | --- | --- | --- | --- | --- | --- | --- | --- | --- | --- | --- | --- |
| MEHHP | **All** | 97.7 | 387 | 20.6 | (17.1) | ND | (ND) | 6.3 | (4.2) | 14.0 | (10.1) | 22.3 | (18.3) | 34.7 | (31.2) | 74.0 | (75.3) | 415.7 | (487.7) | 0.054 | (<0.001) |
|  | ≥7~<18 | 96.9 | 97 | 21.9 | (22.3) | ND | (ND) | 6.2 | (6.4) | 14.9 | (14.6) | 25.8 | (25.1) | 43.9 | (38.7) | 84.7 | (104.4) | 134.7 | (245.9) |  |  |
|  | ≥18~<40 | 97.4 | 73 | 17.9 | (20.8) | ND | (ND) | 4.7 | (3.0) | 12.8 | (12.8) | 17.7 | (23.0) | 31.3 | (36.8) | 62.4 | (84.4) | 327.3 | (487.7) |  |  |
|  | ≥40~<65 | 97.6 | 124 | 18.8 | (14.5) | ND | (ND) | 5.9 | (4.1) | 14.1 | (9.7) | 21.6 | (16.2) | 28.3 | (28.8) | 53.7 | (58.1) | 141.6 | (111.4) |  |  |
|  | ≥65 | 98.9 | 93 | 24.3 | (13.7) | ND | (ND) | 7.4 | (4.1) | 14.0 | (7.8) | 25.2 | (12.8) | 38.9 | (22.7) | 99.5 | (75.3) | 415.7 | (325.4) |  |  |
|  | **Males** | 96.8 | 188 | 18.9 | (18.5) | ND | (ND) | 5.8 | (4.0) | 13.2 | (10.7) | 20.9 | (22.4) | 35.4 | (35.9) | 72.2 | (87.3) | 266.7 | (325.4) | 0.024 | (<0.001) |
|  | ≥7~<18 | 98.1 | 54 | 25.0 | (29.8) | ND | (ND) | 6.2 | (6.7) | 14.8 | (19.3) | 26.8 | (29.2) | 50.3 | (57.3) | 89.0 | (162.1) | 134.7 | (245.9) |  |  |
|  | ≥18~<40 | 97.1 | 33 | 15.4 | (20.2) | ND | (ND) | 3.4 | (2.3) | 10.1 | (14.6) | 16.3 | (23.9) | 29.6 | (37.8) | 67.0 | (84.4) | 94.9 | (105.5) |  |  |
|  | ≥40~<65 | 94.3 | 53 | 15.0 | (14.3) | ND | (ND) | ND | (ND) | 14.4 | (10.6) | 20.7 | (20.2) | 27.6 | (31.6) | 49.5 | (61.6) | 57.6 | (71.3) |  |  |
|  | ≥65 | 98 | 48 | 20.3 | (13.6) | ND | (ND) | 6.6 | (4.0) | 12.3 | (6.9) | 18.6 | (12.4) | 36.3 | (26.9) | 109.6 | (170.0) | 266.7 | (325.4) |  |  |
|  | **Females** | 98.5 | 199 | 22.4 | (15.8) | ND | (ND) | 7.4 | (4.8) | 14.7 | (10.0) | 23.7 | (16.0) | 33.7 | (28.2) | 79.5 | (63.8) | 415.7 | (487.7) | 0.16 | (0.008) |
|  | ≥7~<18 | 95.5 | 43 | 18.5 | (15.5) | ND | (ND) | 1.8 | (0.8) | 14.8 | (10.0) | 19.1 | (20.0) | 37.6 | (28.3) | 69.9 | (64.0) | 101.3 | (91.0) |  |  |
|  | ≥18~<40 | 97.7 | 40 | 20.3 | (21.3) | ND | (ND) | 4.8 | (2.7) | 14.3 | (12.2) | 21.0 | (21.4) | 33.3 | (36.4) | 78.2 | (104.3) | 327.3 | (487.7) |  |  |
|  | ≥40~<65 | 100 | 71 | 22.3 | (14.6) | 5.6 | (3.4) | 7.7 | (4.7) | 13.5 | (9.5) | 22.3 | (14.7) | 30.5 | (21.8) | 60.1 | (58.3) | 141.6 | (111.4) |  |  |
|  | ≥65 | 100 | 45 | 29.4 | (13.9) | 5.3 | (4.8) | 10.1 | (4.9) | 17.3 | (9.4) | 27.3 | (13.2) | 46.6 | (21.4) | 100.8 | (37.1) | 415.7 | (55.2) |  |  |
| MECPP | **All** | 96.2 | 387 | 23.7 | (19.6) | ND | (ND) | 6.9 | (4.0) | 16.8 | (12.1) | 26.2 | (22.5) | 44.1 | (36.3) | 103.7 | (96.5) | 654.4 | (975.1) | 0.002 | (<0.001) |
|  | ≥7~<18 | 96.9 | 97 | 28.3 | (28.9) | ND | (ND) | 8.1 | (5.5) | 19.0 | (19.0) | 30.8 | (34.6) | 56.2 | (56.0) | 109.1 | (123.7) | 150.6 | (339.0) |  |  |
|  | ≥18~<40 | 92.3 | 73 | 15.6 | (18.2) | ND | (ND) | ND | (ND) | 14.3 | (15.4) | 19.7 | (24.9) | 33.1 | (36.3) | 108.0 | (110.2) | 654.4 | (975.1) |  |  |
|  | ≥40~<65 | 96 | 124 | 22.4 | (17.3) | ND | (ND) | 7.1 | (4.2) | 17.2 | (12.1) | 27.2 | (20.2) | 39.2 | (32.5) | 86.0 | (71.5) | 189.2 | (185.4) |  |  |
|  | ≥65 | 98.9 | 93 | 29.2 | (16.5) | ND | (ND) | 7.2 | (5.2) | 17.7 | (8.8) | 26.7 | (15.5) | 43.4 | (29.6) | 178.8 | (87.8) | 434.2 | (529.7) |  |  |
|  | **Males** | 95.8 | 188 | 21.6 | (21.2) | ND | (ND) | 5.7 | (3.3) | 15.8 | (12.3) | 25.4 | (25.6) | 40.1 | (39.2) | 98.5 | (109.0) | 434.2 | (529.7) | 0.006 | (<0.001) |
|  | ≥7~<18 | 98.1 | 54 | 32.1 | (38.2) | ND | (ND) | 7.9 | (9.5) | 19.4 | (23.5) | 32.4 | (37.9) | 62.9 | (71.6) | 118.2 | (159.0) | 150.6 | (339.0) |  |  |
|  | ≥18~<40 | 94.1 | 33 | 15.1 | (19.8) | ND | (ND) | ND | (ND) | 10.9 | (17.8) | 22.2 | (27.3) | 30.2 | (36.4) | 75.6 | (90.3) | 106.6 | (123.7) |  |  |
|  | ≥40~<65 | 92.5 | 53 | 16.7 | (16.0) | ND | (ND) | ND | (ND) | 13.8 | (12.4) | 25.6 | (21.2) | 35.3 | (34.5) | 74.3 | (73.7) | 89.8 | (93.3) |  |  |
|  | ≥65 | 98 | 48 | 23.4 | (15.7) | ND | (ND) | 7.0 | (3.6) | 15.9 | (7.8) | 25.1 | (13.1) | 35.6 | (31.4) | 148.9 | (239.8) | 434.2 | (529.7) |  |  |
|  | **Females** | 96.6 | 199 | 25.8 | (18.2) | ND | (ND) | 8.1 | (3.9) | 17.2 | (11.7) | 30.0 | (20.2) | 47.2 | (32.6) | 107.1 | (84.2) | 654.4 | (975.1) | 0.031 | (0.113) |
|  | ≥7~<18 | 95.5 | 43 | 24.2 | (20.4) | ND | (ND) | 2.0 | (1.3) | 16.9 | (15.4) | 30.6 | (26.1) | 47.2 | (37.8) | 94.6 | (82.1) | 135.3 | (166.4) |  |  |
|  | ≥18~<40 | 90.9 | 40 | 16.1 | (17.0) | ND | (ND) | ND | (ND) | 15.1 | (14.8) | 19.2 | (24.7) | 35.0 | (36.1) | 139.5 | (140.5) | 654.4 | (975.1) |  |  |
|  | ≥40~<65 | 98.6 | 71 | 27.8 | (18.2) | ND | (ND) | 8.8 | (5.6) | 19.0 | (10.2) | 31.2 | (20.1) | 42.8 | (29.9) | 90.0 | (69.3) | 189.2 | (185.4) |  |  |
|  | ≥65 | 100 | 45 | 37.1 | (17.5) | 7.0 | (2.6) | 10.1 | (6.0) | 19.7 | (10.9) | 31.9 | (15.7) | 71.8 | (27.2) | 179.0 | (64.1) | 309.3 | (67.9) |  |  |

| Phthalate metabolites | Group | Detectable rate (%) | *n* | GM | | Min | | 5th | | 25th | | 50th | | 75th | | 95th | | Max | | *P*-value ^b^ | |
| --- | --- | --- | --- | --- | --- | --- | --- | --- | --- | --- | --- | --- | --- | --- | --- | --- | --- | --- | --- | --- | --- |
| MCMHP | **All** | 67.5 | 387 | 2.2 | (1.8) | ND | (ND) | ND | (ND) | ND | (ND) | 4.3 | (3.7) | 8.5 | (7.3) | 17.3 | (17.7) | 167.0 | (203.7) | 0.085 | (<0.001) |
|  | ≥7~<18 | 75.5 | 97 | 2.9 | (2.9) | ND | (ND) | ND | (ND) | 0.9 | (1.2) | 4.6 | (5.6) | 10.9 | (10.1) | 18.7 | (24.5) | 37.5 | (46.2) |  |  |
|  | ≥18~<40 | 73.1 | 73 | 2.0 | (2.3) | ND | (ND) | ND | (ND) | ND | (ND) | 4.1 | (4.9) | 6.4 | (9.6) | 14.7 | (16.7) | 115.1 | (171.4) |  |  |
|  | ≥40~<65 | 62.1 | 124 | 1.8 | (1.4) | ND | (ND) | ND | (ND) | ND | (ND) | 3.9 | (2.8) | 6.9 | (6.1) | 14.4 | (13.6) | 29.4 | (30.8) |  |  |
|  | ≥65 | 61.7 | 93 | 2.4 | (1.4) | ND | (ND) | ND | (ND) | ND | (ND) | 4.3 | (2.9) | 8.9 | (5.3) | 27.1 | (16.0) | 167.0 | (203.7) |  |  |
|  | **Males** | 70.5 | 188 | 2.3 | (2.3) | ND | (ND) | ND | (ND) | ND | (ND) | 4.4 | (4.8) | 8.6 | (8.8) | 16.4 | (20.8) | 167.0 | (203.7) | 0.082 | (0.003) |
|  | ≥7~<18 | 81.5 | 54 | 3.4 | (4.1) | ND | (ND) | ND | (ND) | 2.0 | (3.1) | 6.0 | (6.3) | 11.7 | (11.8) | 18.6 | (31.9) | 24.0 | (41.1) |  |  |
|  | ≥18~<40 | 73.5 | 33 | 1.8 | (2.3) | ND | (ND) | ND | (ND) | ND | (ND) | 3.7 | (4.7) | 5.8 | (8.3) | 13.1 | (14.7) | 13.3 | (16.4) |  |  |
|  | ≥40~<65 | 73.6 | 53 | 2.4 | (2.3) | ND | (ND) | ND | (ND) | ND | (ND) | 4.7 | (5.1) | 7.6 | (8.7) | 13.7 | (14.5) | 15.0 | (16.0) |  |  |
|  | ≥65 | 53.1 | 48 | 1.7 | (1.2) | ND | (ND) | ND | (ND) | ND | (ND) | 2.4 | (2.6) | 8.3 | (6.5) | 43.6 | (69.1) | 167.0 | (203.7) |  |  |
|  | **Females** | 64.7 | 199 | 2.1 | (1.5) | ND | (ND) | ND | (ND) | ND | (ND) | 4.2 | (3.1) | 8.4 | (6.1) | 20.4 | (14.7) | 115.1 | (171.4) | 0.03 | (0.010) |
|  | ≥7~<18 | 68.2 | 43 | 2.3 | (1.9) | ND | (ND) | ND | (ND) | ND | (ND) | 4.4 | (3.9) | 9.9 | (7.8) | 28.8 | (22.8) | 37.5 | (46.2) |  |  |
|  | ≥18~<40 | 72.7 | 40 | 2.3 | (2.4) | ND | (ND) | ND | (ND) | ND | (ND) | 4.6 | (5.0) | 6.7 | (10.3) | 16.2 | (18.8) | 115.1 | (171.4) |  |  |
|  | ≥40~<65 | 53.5 | 71 | 1.4 | (0.9) | ND | (ND) | ND | (ND) | ND | (ND) | 2.1 | (2.2) | 6.4 | (4.2) | 16.2 | (10.8) | 29.4 | (30.8) |  |  |
|  | ≥65 | 71.1 | 45 | 3.5 | (1.6) | ND | (ND) | ND | (ND) | ND | (ND) | 6.2 | (3.2) | 11.2 | (5.2) | 30.2 | (12.0) | 34.9 | (12.6) |  |  |
| MnBP | **All** | 88.6 | 387 | 13.9 | (11.5) | ND | (ND) | ND | (ND) | 9.6 | (7.7) | 19.8 | (17.3) | 34.3 | (32.1) | 132.4 | (109.5) | 5088.2 | (6105.8) | 0.044 | (<0.001) |
|  | ≥7~<18 | 92.9 | 97 | 17.6 | (18.0) | ND | (ND) | ND | (ND) | 11.2 | (13.8) | 23.6 | (21.7) | 40.6 | (43.5) | 109.2 | (125.8) | 194.0 | (158.2) |  |  |
|  | ≥18~<40 | 89.7 | 73 | 11.0 | (12.8) | ND | (ND) | ND | (ND) | 9.9 | (9.9) | 16.6 | (20.4) | 23.7 | (31.5) | 68.9 | (83.0) | 497.0 | (1515.8) |  |  |
|  | ≥40~<65 | 87.9 | 124 | 13.3 | (10.2) | ND | (ND) | ND | (ND) | 8.7 | (6.3) | 19.9 | (15.1) | 33.6 | (30.2) | 132.4 | (100.9) | 1780.2 | (836.7) |  |  |
|  | ≥65 | 84 | 93 | 13.8 | (7.8) | ND | (ND) | ND | (ND) | 7.4 | (4.2) | 18.3 | (11.2) | 41.0 | (24.8) | 306.4 | (106.1) | 5088.2 | (6105.8) |  |  |
|  | **Males** | 87.9 | 188 | 12.0 | (11.8) | ND | (ND) | ND | (ND) | 7.6 | (7.1) | 16.5 | (17.8) | 30.5 | (34.1) | 134.8 | (121.5) | 5088.2 | (6105.8) | 0.012 | (<0.001) |
|  | ≥7~<18 | 88.9 | 54 | 16.4 | (19.5) | ND | (ND) | ND | (ND) | 10.9 | (15.1) | 25.1 | (29.4) | 53.6 | (61.3) | 146.2 | (139.6) | 194.0 | (158.2) |  |  |
|  | ≥18~<40 | 88.2 | 33 | 9.3 | (12.1) | ND | (ND) | ND | (ND) | 7.3 | (7.1) | 13.7 | (19.8) | 20.3 | (30.7) | 175.1 | (501.3) | 497.0 | (1515.8) |  |  |
|  | ≥40~<65 | 90.6 | 53 | 12.2 | (11.6) | ND | (ND) | ND | (ND) | 8.6 | (7.9) | 17.2 | (14.5) | 27.6 | (32.6) | 103.4 | (92.7) | 480.7 | (405.1) |  |  |
|  | ≥65 | 83.7 | 48 | 10.0 | (6.7) | ND | (ND) | ND | (ND) | 4.9 | (3.3) | 10.7 | (8.9) | 30.4 | (18.4) | 546.8 | (174.0) | 5088.2 | (6105.8) |  |  |
|  | **Females** | 89.2 | 199 | 16.0 | (11.3) | ND | (ND) | ND | (ND) | 11.2 | (8.0) | 22.1 | (16.6) | 38.1 | (31.0) | 132.5 | (78.6) | 1780.2 | (836.7) | 0.111 | (0.339) |
|  | ≥7~<18 | 97.7 | 43 | 19.3 | (16.2) | ND | (ND) | 5.7 | (3.0) | 13.3 | (12.9) | 21.0 | (16.0) | 33.1 | (32.0) | 76.8 | (58.5) | 108.0 | (78.6) |  |  |
|  | ≥18~<40 | 90.9 | 40 | 12.7 | (13.4) | ND | (ND) | ND | (ND) | 10.9 | (11.5) | 19.6 | (21.1) | 26.1 | (32.0) | 78.2 | (115.2) | 88.4 | (122.9) |  |  |
|  | ≥40~<65 | 85.9 | 71 | 14.2 | (9.3) | ND | (ND) | ND | (ND) | 8.8 | (4.6) | 21.1 | (15.6) | 40.4 | (28.0) | 144.6 | (143.4) | 1780.2 | (836.7) |  |  |
|  | ≥65 | 84.4 | 45 | 19.6 | (9.2) | ND | (ND) | ND | (ND) | 15.4 | (6.3) | 26.6 | (15.8) | 55.3 | (29.0) | 317.7 | (143.9) | 540.9 | (589.6) |  |  |

| Phthalate metabolites | Group | Detectable rate (%) | *n* | GM | | Min | | 5th | | 25th | | 50th | | 75th | | 95th | | Max | | *P*-value ^b^ | |
| --- | --- | --- | --- | --- | --- | --- | --- | --- | --- | --- | --- | --- | --- | --- | --- | --- | --- | --- | --- | --- | --- |
| MiBP | **All** | 73.4 | 387 | 5.0 | (4.1) | ND | (ND) | ND | (ND) | ND | (ND) | 10.4 | (8.8) | 20.1 | (19.6) | 69.7 | (63.0) | 138.0 | (286.6) | 0.156 | (<0.001) |
|  | ≥7~<18 | 80.6 | 97 | 7.2 | (7.3) | ND | (ND) | ND | (ND) | 4.7 | (4.9) | 13.6 | (15.3) | 24.2 | (29.3) | 76.4 | (75.0) | 138.0 | (200.1) |  |  |
|  | ≥18~<40 | 78.2 | 73 | 5.1 | (5.9) | ND | (ND) | ND | (ND) | 1.5 | (3.4) | 10.6 | (11.3) | 18.8 | (23.1) | 79.2 | (121.6) | 129.7 | (286.6) |  |  |
|  | ≥40~<65 | 71 | 124 | 4.5 | (3.5) | ND | (ND) | ND | (ND) | ND | (ND) | 10.2 | (6.9) | 19.5 | (18.7) | 53.1 | (42.7) | 115.0 | (87.4) |  |  |
|  | ≥65 | 64.9 | 93 | 3.8 | (2.2) | ND | (ND) | ND | (ND) | ND | (ND) | 7.1 | (4.5) | 18.8 | (11.2) | 86.3 | (33.4) | 110.9 | (36.1) |  |  |
|  | **Males** | 74.2 | 188 | 4.6 | (4.5) | ND | (ND) | ND | (ND) | ND | (ND) | 10.0 | (9.3) | 19.1 | (20.8) | 58.0 | (64.9) | 138.0 | (286.6) | 0.012 | (<0.001) |
|  | ≥7~<18 | 83.3 | 54 | 7.8 | (9.3) | ND | (ND) | ND | (ND) | 6.1 | (6.6) | 15.0 | (18.2) | 23.6 | (29.7) | 72.7 | (78.3) | 138.0 | (200.1) |  |  |
|  | ≥18~<40 | 82.4 | 33 | 6.3 | (8.2) | ND | (ND) | ND | (ND) | 2.7 | (5.0) | 11.6 | (14.3) | 21.1 | (30.9) | 83.6 | (190.5) | 129.7 | (286.6) |  |  |
|  | ≥40~<65 | 67.9 | 53 | 3.2 | (3.1) | ND | (ND) | ND | (ND) | ND | (ND) | 6.6 | (6.0) | 16.0 | (17.0) | 54.6 | (65.5) | 72.1 | (72.3) |  |  |
|  | ≥65 | 65.3 | 48 | 3.1 | (2.0) | ND | (ND) | ND | (ND) | ND | (ND) | 6.8 | (4.2) | 14.2 | (10.1) | 50.3 | (29.8) | 72.9 | (35.9) |  |  |
|  | **Females** | 72.5 | 199 | 5.4 | (3.8) | ND | (ND) | ND | (ND) | ND | (ND) | 11.0 | (8.6) | 23.8 | (19.0) | 83.8 | (53.0) | 115.0 | (141.7) | 0.643 | (0.171) |
|  | ≥7~<18 | 77.3 | 43 | 6.4 | (5.4) | ND | (ND) | ND | (ND) | 3.0 | (2.1) | 10.4 | (10.3) | 27.7 | (29.2) | 80.7 | (79.3) | 100.0 | (83.3) |  |  |
|  | ≥18~<40 | 75 | 40 | 4.3 | (4.5) | ND | (ND) | ND | (ND) | ND | (ND) | 9.4 | (8.9) | 16.5 | (21.4) | 98.3 | (104.8) | 101.9 | (141.7) |  |  |
|  | ≥40~<65 | 73.2 | 71 | 5.9 | (3.8) | ND | (ND) | ND | (ND) | ND | (ND) | 12.9 | (7.0) | 24.8 | (19.6) | 61.2 | (39.5) | 115.0 | (87.4) |  |  |
|  | ≥65 | 64.4 | 45 | 4.8 | (2.3) | ND | (ND) | ND | (ND) | ND | (ND) | 8.9 | (5.2) | 26.8 | (12.8) | 106.0 | (35.2) | 110.9 | (36.1) |  |  |
| MEP | **All** | 91.6 | 387 | 13.3 | (11.0) | ND | (ND) | ND | (ND) | 6.6 | (5.2) | 14.0 | (12.5) | 32.0 | (29.2) | 162.5 | (175.3) | 4675.1 | (3286.0) | 0.166 | (<0.001) |
|  | ≥7~<18 | 92.9 | 97 | 11.5 | (11.7) | ND | (ND) | ND | (ND) | 6.5 | (5.5) | 12.4 | (14.2) | 25.3 | (29.6) | 80.8 | (151.3) | 412.0 | (626.4) |  |  |
|  | ≥18~<40 | 98.7 | 73 | 19.9 | (23.1) | ND | (ND) | 3.3 | (3.3) | 9.2 | (9.5) | 19.7 | (22.1) | 42.7 | (41.0) | 258.4 | (461.5) | 2376.0 | (3286.0) |  |  |
|  | ≥40~<65 | 92.7 | 124 | 13.5 | (10.4) | ND | (ND) | ND | (ND) | 6.6 | (5.2) | 14.0 | (10.1) | 30.7 | (22.9) | 172.6 | (106.9) | 1919.0 | (1017.1) |  |  |
|  | ≥65 | 83 | 93 | 11.1 | (6.3) | ND | (ND) | ND | (ND) | 4.8 | (3.3) | 12.7 | (8.1) | 37.0 | (23.9) | 183.0 | (130.0) | 4675.1 | (2571.3) |  |  |
|  | **Males** | 92.1 | 188 | 10.7 | (10.5) | ND | (ND) | ND | (ND) | 5.7 | (4.4) | 11.5 | (12.2) | 26.7 | (29.1) | 113.4 | (174.9) | 2376.0 | (3286.0) | 0.016 | (<0.001) |
|  | ≥7~<18 | 96.3 | 54 | 12.9 | (15.3) | ND | (ND) | 1.3 | (1.8) | 8.2 | (9.3) | 13.0 | (17.9) | 24.8 | (33.2) | 72.8 | (155.9) | 248.6 | (392.3) |  |  |
|  | ≥18~<40 | 100 | 33 | 20.0 | (26.2) | 2.4 | (3.3) | 3.3 | (3.3) | 8.7 | (9.1) | 18.7 | (22.8) | 41.0 | (48.1) | 811.9 | (1176.9) | 2376.0 | (3286.0) |  |  |
|  | ≥40~<65 | 88.7 | 53 | 6.9 | (6.6) | ND | (ND) | ND | (ND) | 3.5 | (3.5) | 9.0 | (8.6) | 18.2 | (17.9) | 84.7 | (68.7) | 447.5 | (407.2) |  |  |
|  | ≥65 | 85.7 | 48 | 9.1 | (6.1) | ND | (ND) | ND | (ND) | 4.7 | (3.0) | 9.5 | (6.8) | 33.1 | (19.1) | 168.3 | (203.4) | 444.9 | (219.7) |  |  |
|  | **Females** | 91.2 | 199 | 16.4 | (11.5) | ND | (ND) | ND | (ND) | 7.6 | (5.4) | 17.9 | (13.4) | 41.3 | (29.2) | 201.1 | (209.3) | 4675.1 | (2571.3) | 0.095 | (0.029) |
|  | ≥7~<18 | 88.6 | 43 | 10.0 | (8.4) | ND | (ND) | ND | (ND) | 5.0 | (3.5) | 11.1 | (9.7) | 26.9 | (27.0) | 222.3 | (204.5) | 412.0 | (626.4) |  |  |
|  | ≥18~<40 | 97.7 | 40 | 19.9 | (20.9) | ND | (ND) | 2.6 | (2.5) | 9.6 | (9.6) | 20.8 | (21.7) | 42.9 | (36.2) | 465.5 | (596.2) | 703.0 | (1258.4) |  |  |
|  | ≥40~<65 | 95.8 | 71 | 22.2 | (14.6) | ND | (ND) | 2.0 | (1.4) | 10.9 | (6.1) | 21.2 | (12.9) | 42.2 | (37.7) | 267.1 | (211.6) | 1919.0 | (1017.1) |  |  |
|  | ≥65 | 80 | 45 | 13.6 | (6.4) | ND | (ND) | ND | (ND) | 6.1 | (3.3) | 17.8 | (9.4) | 49.5 | (25.2) | 200.7 | (102.2) | 4675.1 | (2571.3) |  |  |

| Phthalate metabolites | Group | Detectable rate (%) | *n* | GM | | Min | | 5th | | 25th | | 50th | | 75th | | 95th | | Max | | *P*-value ^b^ | |
| --- | --- | --- | --- | --- | --- | --- | --- | --- | --- | --- | --- | --- | --- | --- | --- | --- | --- | --- | --- | --- | --- |
| MiNP | **All** | 12.7 | 387 | ND | (ND) | ND | (ND) | ND | (ND) | ND | (ND) | ND | (ND) | ND | (ND) | 4.9 | (3.7) | 43.4 | (49.5) | <0.001 | (0.216) |
|  | ≥7~<18 | 12.2 | 97 | ND | (ND) | ND | (ND) | ND | (ND) | ND | (ND) | ND | (ND) | ND | (ND) | 9.1 | (8.1) | 43.4 | (49.5) |  |  |
|  | ≥18~<40 | 20.5 | 73 | 0.2 | (0.3) | ND | (ND) | ND | (ND) | ND | (ND) | ND | (ND) | ND | (ND) | 4.5 | (5.2) | 12.1 | (15.0) |  |  |
|  | ≥40~<65 | 6.5 | 124 | ND | (ND) | ND | (ND) | ND | (ND) | ND | (ND) | ND | (ND) | ND | (ND) | 2.9 | (2.9) | 12.4 | (8.5) |  |  |
|  | ≥65 | 14.9 | 93 | ND | (ND) | ND | (ND) | ND | (ND) | ND | (ND) | ND | (ND) | ND | (ND) | 6.0 | (3.4) | 39.7 | (11.1) |  |  |
|  | **Males** | 10.5 | 188 | ND | (ND) | ND | (ND) | ND | (ND) | ND | (ND) | ND | (ND) | ND | (ND) | 4.1 | (4.0) | 43.4 | (49.5) | <0.001 | (0.164) |
|  | ≥7~<18 | 13 | 54 | 0.2 | (0.3) | ND | (ND) | ND | (ND) | ND | (ND) | ND | (ND) | ND | (ND) | 10.2 | (12.1) | 43.4 | (49.5) |  |  |
|  | ≥18~<40 | 14.7 | 33 | ND | (ND) | ND | (ND) | ND | (ND) | ND | (ND) | ND | (ND) | ND | (ND) | 8.1 | (12.2) | 12.1 | (15.0) |  |  |
|  | ≥40~<65 | 1.9 | 53 | ND | (ND) | ND | (ND) | ND | (ND) | ND | (ND) | ND | (ND) | ND | (ND) | ND | (ND) | 2.4 | (3.7) |  |  |
|  | ≥65 | 14.3 | 48 | ND | (ND) | ND | (ND) | ND | (ND) | ND | (ND) | ND | (ND) | ND | (ND) | 3.8 | (3.4) | 5.6 | (5.3) |  |  |
|  | **Females** | 14.7 | 199 | ND | (ND) | ND | (ND) | ND | (ND) | ND | (ND) | ND | (ND) | ND | (ND) | 5.5 | (3.6) | 39.7 | (22.2) | <0.001 | (0.638) |
|  | ≥7~<18 | 11.4 | 43 | ND | (ND) | ND | (ND) | ND | (ND) | ND | (ND) | ND | (ND) | ND | (ND) | 13.2 | (9.9) | 16.9 | (22.2) |  |  |
|  | ≥18~<40 | 25 | 40 | 0.2 | (0.3) | ND | (ND) | ND | (ND) | ND | (ND) | ND | (ND) | ND | (ND) | 3.2 | (3.6) | 3.5 | (5.2) |  |  |
|  | ≥40~<65 | 9.9 | 71 | ND | (ND) | ND | (ND) | ND | (ND) | ND | (ND) | ND | (ND) | ND | (ND) | 5.5 | (3.6) | 12.4 | (8.5) |  |  |
|  | ≥65 | 15.6 | 45 | ND | (ND) | ND | (ND) | ND | (ND) | ND | (ND) | ND | (ND) | ND | (ND) | 24.4 | (3.5) | 39.7 | (11.1) |  |  |
| MBzP | **All** | 23.9 | 387 | 0.4 | (0.3) | ND | (ND) | ND | (ND) | ND | (ND) | ND | (ND) | ND | (ND) | 7.4 | (5.9) | 75.9 | (27.1) | <0.001 | (0.291) |
|  | ≥7~<18 | 28.6 | 97 | 0.4 | (0.4) | ND | (ND) | ND | (ND) | ND | (ND) | ND | (ND) | 1.7 | (2.1) | 7.5 | (12.4) | 12.7 | (27.1) |  |  |
|  | ≥18~<40 | 30.8 | 73 | 0.3 | (0.4) | ND | (ND) | ND | (ND) | ND | (ND) | ND | (ND) | 1.4 | (2.3) | 5.2 | (5.7) | 16.7 | (9.3) |  |  |
|  | ≥40~<65 | 17.7 | 124 | 0.4 | (0.3) | ND | (ND) | ND | (ND) | ND | (ND) | ND | (ND) | ND | (ND) | 7.6 | (5.8) | 43.7 | (15.8) |  |  |
|  | ≥65 | 21.3 | 93 | 0.5 | (0.3) | ND | (ND) | ND | (ND) | ND | (ND) | ND | (ND) | ND | (ND) | 8.2 | (4.8) | 75.9 | (8.5) |  |  |
|  | **Males** | 26.8 | 188 | 0.4 | (0.4) | ND | (ND) | ND | (ND) | ND | (ND) | ND | (ND) | 1.4 | (2.0) | 6.1 | (7.7) | 16.7 | (27.1) | 0.017 | (0.092) |
|  | ≥7~<18 | 38.9 | 54 | 0.5 | (0.6) | ND | (ND) | ND | (ND) | ND | (ND) | ND | (ND) | 3.0 | (3.0) | 11.1 | (15.2) | 12.7 | (27.1) |  |  |
|  | ≥18~<40 | 26.5 | 33 | 0.3 | (0.3) | ND | (ND) | ND | (ND) | ND | (ND) | ND | (ND) | 1.1 | (1.2) | 7.0 | (6.9) | 16.7 | (9.3) |  |  |
|  | ≥40~<65 | 18.9 | 53 | 0.3 | (0.3) | ND | (ND) | ND | (ND) | ND | (ND) | ND | (ND) | ND | (ND) | 5.1 | (6.9) | 10.0 | (12.8) |  |  |
|  | ≥65 | 22.4 | 48 | 0.5 | (0.3) | ND | (ND) | ND | (ND) | ND | (ND) | ND | (ND) | ND | (ND) | 7.5 | (5.7) | 12.6 | (8.5) |  |  |
|  | **Females** | 21.1 | 199 | 0.4 | (0.3) | ND | (ND) | ND | (ND) | ND | (ND) | ND | (ND) | ND | (ND) | 8.2 | (5.1) | 75.9 | (18.3) | 0.001 | (0.467) |
|  | ≥7~<18 | 15.9 | 43 | ND | (ND) | ND | (ND) | ND | (ND) | ND | (ND) | ND | (ND) | ND | (ND) | 6.4 | (5.2) | 10.2 | (18.3) |  |  |
|  | ≥18~<40 | 34.1 | 40 | 0.4 | (0.4) | ND | (ND) | ND | (ND) | ND | (ND) | ND | (ND) | 1.5 | (2.5) | 7.1 | (5.6) | 9.0 | (8.1) |  |  |
|  | ≥40~<65 | 16.9 | 71 | 0.4 | (0.3) | ND | (ND) | ND | (ND) | ND | (ND) | ND | (ND) | ND | (ND) | 9.3 | (5.4) | 43.7 | (15.8) |  |  |
|  | ≥65 | 20 | 45 | 0.6 | (0.3) | ND | (ND) | ND | (ND) | ND | (ND) | ND | (ND) | ND | (ND) | 9.5 | (4.6) | 75.9 | (6.8) |  |  |

| Phthalate metabolites | Group | Detectable rate (%) | *n* | GM | | Min | | 5th | | 25th | | 50th | | 75th | | 95th | | Max | | *P*-value ^b^ | |
| --- | --- | --- | --- | --- | --- | --- | --- | --- | --- | --- | --- | --- | --- | --- | --- | --- | --- | --- | --- | --- | --- |
| MMP | **All** | 97 | 387 | 32.7 | (27.1) | ND | (ND) | 5.1 | (4.1) | 16.6 | (12.6) | 32.7 | (26.7) | 69.3 | (63.9) | 283.4 | (311.6) | 6529.6 | (7117.3) | <0.001 | (0.001) |
|  | ≥7~<18 | 98 | 97 | 37.5 | (38.2) | ND | (ND) | 5.1 | (4.8) | 21.2 | (18.1) | 41.2 | (43.0) | 79.0 | (83.3) | 215.7 | (381.8) | 1355.1 | (1004.9) |  |  |
|  | ≥18~<40 | 98.7 | 73 | 25.3 | (29.4) | ND | (ND) | 6.6 | (6.7) | 12.9 | (13.8) | 23.9 | (26.7) | 41.0 | (55.8) | 175.0 | (291.2) | 538.5 | (780.9) |  |  |
|  | ≥40~<65 | 95.2 | 124 | 24.9 | (19.1) | ND | (ND) | 1.1 | (0.6) | 11.6 | (8.4) | 25.2 | (18.8) | 56.0 | (53.3) | 248.1 | (276.0) | 6216.1 | (3660.6) |  |  |
|  | ≥65 | 96.8 | 93 | 50.1 | (28.3) | ND | (ND) | 9.0 | (4.7) | 28.5 | (13.0) | 46.4 | (27.6) | 105.9 | (58.5) | 391.1 | (417.1) | 6529.6 | (7117.3) |  |  |
|  | **Males** | 97.9 | 188 | 31.5 | (31.0) | ND | (ND) | 4.8 | (4.6) | 16.3 | (14.3) | 32.1 | (32.0) | 66.9 | (69.4) | 235.6 | (330.6) | 993.9 | (1350.3) | <0.001 | (0.015) |
|  | ≥7~<18 | 100 | 54 | 37.4 | (44.5) | 4.5 | (4.5) | 5.2 | (8.2) | 20.9 | (22.9) | 41.6 | (45.9) | 70.4 | (83.7) | 138.5 | (392.7) | 159.0 | (456.4) |  |  |
|  | ≥18~<40 | 100 | 33 | 20.8 | (27.2) | 3.7 | (5.3) | 5.4 | (6.0) | 12.4 | (13.8) | 19.4 | (25.9) | 28.5 | (37.0) | 253.4 | (472.0) | 538.5 | (780.9) |  |  |
|  | ≥40~<65 | 94.3 | 53 | 21.0 | (20.1) | ND | (ND) | ND | (ND) | 11.0 | (8.0) | 23.7 | (22.9) | 41.4 | (52.7) | 259.8 | (307.0) | 993.9 | (924.3) |  |  |
|  | ≥65 | 98 | 48 | 54.1 | (36.3) | ND | (ND) | 8.3 | (6.4) | 31.9 | (13.8) | 48.3 | (37.6) | 107.2 | (79.5) | 391.1 | (435.7) | 865.6 | (1350.3) |  |  |
|  | **Females** | 96.1 | 199 | 33.9 | (23.9) | ND | (ND) | 5.1 | (3.1) | 17.2 | (10.1) | 32.9 | (23.2) | 75.4 | (61.7) | 291.3 | (282.5) | 6529.6 | (7117.3) | 0.111 | (0.045) |
|  | ≥7~<18 | 95.5 | 43 | 37.5 | (31.5) | ND | (ND) | 1.3 | (0.7) | 21.2 | (11.5) | 39.3 | (40.0) | 81.1 | (82.3) | 1088.8 | (667.6) | 1355.1 | (1004.9) |  |  |
|  | ≥18~<40 | 97.7 | 40 | 29.8 | (31.4) | ND | (ND) | 6.9 | (6.8) | 14.9 | (14.0) | 27.0 | (32.2) | 57.2 | (65.1) | 177.4 | (267.4) | 515.6 | (526.2) |  |  |
|  | ≥40~<65 | 95.8 | 71 | 28.2 | (18.5) | ND | (ND) | 3.2 | (1.4) | 11.5 | (8.7) | 29.8 | (14.3) | 66.1 | (54.0) | 289.8 | (253.3) | 6216.1 | (3660.6) |  |  |
|  | ≥65 | 95.6 | 45 | 46.2 | (21.8) | ND | (ND) | 3.6 | (1.3) | 21.5 | (11.2) | 43.3 | (22.3) | 99.8 | (47.1) | 2086.7 | (561.2) | 6529.6 | (7117.3) |  |  |

^a^ Abbreviations are listed in the footnote of Table 2; unadjusted level of each phthalate metabolite was shown in the parentheses; ND: not detectable; Detectable rate=number of urine sample with level of each phthalate metabolite above detection limit/ all analyzed urine samples.

^b^ Kruskal Wallis test; p-value for unadjusted level of each phthalate metabolite was shown in the parentheses.
