# Supplementary material for: Age and Gender Differences in Urinary Levels of Eleven Phthalate Metabolites in General Taiwanese Population after a DEHP Episode
Source: PLoS One. 2015 Jul 24;10(7):e0133782. doi: 10.1371/journal.pone.0133782 (PMC4514596; doi:10.1371/journal.pone.0133782)
Supplement: S3 Table — (DOCX) [file pone.0133782.s003.docx]

**S3 Table.** **Distribution of creatinine-adjusted levels (μg/g creatinine or ng/ml) in phthalate metabolites ^a^ from a sample of the general Taiwanese population by different regions (N=387).**

| Phthalate metabolites | Taiwan | Detectable rate (%) | *n* | Min | | 5th | | 25th | | 50th | | 75th | | 95th | | Max | | *P*-value ^b^ | |
| --- | --- | --- | --- | --- | --- | --- | --- | --- | --- | --- | --- | --- | --- | --- | --- | --- | --- | --- | --- |
|  |  |  |  |  |  |  |  |  |  |  |  |  |  |  |  |  |  |  |  |
| MEHP | Northern Taiwan | 60.3 | 121 | ND | (ND) | ND | (ND) | ND | (ND) | 4.7 | (4.7) | 9.6 | (8.2) | 26.5 | (25.4) | 42.9 | (58.5) | <0.001 | (<0.001) |
|  | Central Taiwan | 98.1 | 54 | ND | (ND) | 2.0 | (2.5) | 4.7 | (4.5) | 8.4 | (8.4) | 13.3 | (8.3) | 47.7 | (32.7) | 74.3 | (69.1) |  |  |
|  | Southern Taiwan | 78.4 | 102 | ND | (ND) | ND | (ND) | 4.3 | (3.4) | 11.2 | (11.2) | 19.2 | (16.4) | 49.7 | (42.3) | 110.3 | (138.9) |  |  |
|  | Eastern Taiwan | 76.9 | 65 | ND | (ND) | ND | (ND) | 2.6 | (2.2) | 7.4 | (7.4) | 12.5 | (12.7) | 33.6 | (28.4) | 48.0 | (36.0) |  |  |
|  | Remote | 93.3 | 45 | ND | (ND) | ND | (ND) | 4.2 | (5.3) | 8.8 | (8.8) | 19.3 | (14.6) | 35.4 | (29.2) | 37.2 | (39.7) |  |  |
| MEOHP | Northern Taiwan | 99.2 | 121 | ND | (ND) | 4.4 | (3.4) | 9.6 | (6.4) | 13.4 | (13.4) | 21.1 | (21.0) | 55.1 | (47.8) | 72.8 | (66.3) | <0.001 | (0.003) |
|  | Central Taiwan | 96.3 | 54 | ND | (ND) | 3.1 | (2.8) | 9.3 | (8.2) | 16.9 | (16.9) | 21.7 | (25.1) | 78.3 | (46.6) | 146.5 | (178.8) |  |  |
|  | Southern Taiwan | 100 | 102 | 2.5 | (2.2) | 4.9 | (3.7) | 9.6 | (9.0) | 16.6 | (16.6) | 24.4 | (22.2) | 56.4 | (73.4) | 244.5 | (289.6) |  |  |
|  | Eastern Taiwan | 72.3 | 65 | ND | (ND) | ND | (ND) | ND | (ND) | 9.3 | (9.3) | 18.4 | (17.5) | 40.0 | (54.8) | 65.6 | (98.6) |  |  |
|  | Remote | 100 | 45 | 3.4 | (2.5) | 3.9 | (3.0) | 6.7 | (5.9) | 10.6 | (10.6) | 16.6 | (16.9) | 29.6 | (41.1) | 44.6 | (64.6) |  |  |
| MEHHP | Northern Taiwan | 98.3 | 121 | ND | (ND) | 7.4 | (4.2) | 13.1 | (9.3) | 18.9 | (18.9) | 28.2 | (26.2) | 53.2 | (61.6) | 98.6 | (80.6) | 0.001 | (0.021) |
|  | Central Taiwan | 98.1 | 54 | ND | (ND) | 5.4 | (6.8) | 14.3 | (10.0) | 24.5 | (24.5) | 33.5 | (29.5) | 98.4 | (74.9) | 266.7 | (325.4) |  |  |
|  | Southern Taiwan | 94.1 | 102 | ND | (ND) | ND | (ND) | 14.3 | (11.3) | 25.9 | (25.9) | 39.3 | (33.8) | 95.4 | (105.0) | 415.7 | (487.7) |  |  |
|  | Eastern Taiwan | 100 | 65 | 7.8 | (2.7) | 9.9 | (4.3) | 16.9 | (11.0) | 26.3 | (26.3) | 48.2 | (59.9) | 94.7 | (100.1) | 141.6 | (183.0) |  |  |
|  | Remote | 100 | 45 | 5.3 | (4.1) | 6.2 | (5.1) | 11.0 | (9.6) | 18.3 | (18.3) | 24.9 | (27.1) | 58.8 | (68.9) | 94.9 | (106.1) |  |  |
| MECPP | Northern Taiwan | 100 | 121 | 7.3 | (2.8) | 10.2 | (6.0) | 18.0 | (13.9) | 26.7 | (26.7) | 40.2 | (35.0) | 91.3 | (93.2) | 123.3 | (113.6) | 0.001 | (0.149) |
|  | Central Taiwan | 98.1 | 54 | ND | (ND) | 7.6 | (4.8) | 16.5 | (10.9) | 23.8 | (23.8) | 35.1 | (31.7) | 103.4 | (81.3) | 434.2 | (529.7) |  |  |
|  | Southern Taiwan | 87.3 | 102 | ND | (ND) | ND | (ND) | 16.2 | (10.9) | 32.3 | (32.3) | 63.3 | (55.2) | 140.1 | (162.8) | 654.4 | (975.1) |  |  |
|  | Eastern Taiwan | 98.5 | 65 | ND | (ND) | 8.1 | (6.6) | 19.9 | (15.3) | 30.6 | (30.6) | 49.0 | (37.7) | 104.7 | (103.0) | 179.1 | (215.8) |  |  |
|  | Remote | 100 | 45 | 6.6 | (2.6) | 6.9 | (4.5) | 10.8 | (10.5) | 16.7 | (16.7) | 28.8 | (29.3) | 72.7 | (69.6) | 106.6 | (84.3) |  |  |
| MCMHP | Northern Taiwan | 72.7 | 121 | ND | (ND) | ND | (ND) | ND | (ND) | 5.5 | (5.5) | 9.7 | (7.5) | 17.9 | (17.1) | 37.5 | (46.2) | <0.001 | (<0.001) |
|  | Central Taiwan | 40.7 | 54 | ND | (ND) | ND | (ND) | ND | (ND) | 0.5 | (0.5) | 3.7 | (4.2) | 18.9 | (18.0) | 167.0 | (203.7) |  |  |
|  | Southern Taiwan | 84.3 | 102 | ND | (ND) | ND | (ND) | 3.5 | (2.6) | 5.9 | (5.9) | 10.3 | (8.9) | 22.8 | (23.8) | 115.0 | (171.4) |  |  |
|  | Eastern Taiwan | 53.8 | 65 | ND | (ND) | ND | (ND) | ND | (ND) | 2.2 | (2.2) | 6.7 | (6.8) | 19.9 | (30.7) | 60.2 | (91.4) |  |  |
|  | Remote | 62.2 | 45 | ND | (ND) | ND | (ND) | ND | (ND) | 2.1 | (2.1) | 6.4 | (6.1) | 11.2 | (15.1) | 13.0 | (18.9) |  |  |
| MnBP | Northern Taiwan | 81 | 121 | ND | (ND) | ND | (ND) | 8.8 | (4.7) | 19.8 | (19.8) | 33.6 | (30.2) | 128.3 | (142.8) | 5088.2 | (6105.8) | 0.638 | (0.503) |
|  | Central Taiwan | 92.6 | 54 | ND | (ND) | ND | (ND) | 10.6 | (8.1) | 18.5 | (18.5) | 29.4 | (29.7) | 79.9 | (88.2) | 206.2 | (136.9) |  |  |
|  | Southern Taiwan | 89.2 | 102 | ND | (ND) | ND | (ND) | 10.1 | (8.2) | 21.3 | (21.3) | 37.3 | (37.9) | 149.6 | (128.1) | 497.0 | (1515.8) |  |  |
|  | Eastern Taiwan | 92.3 | 65 | ND | (ND) | ND | (ND) | 11.2 | (9.3) | 20.6 | (20.6) | 33.6 | (33.5) | 82.8 | (92.6) | 161.9 | (122.9) |  |  |
|  | Remote | 95.6 | 45 | ND | (ND) | 0.9 | (0.8) | 5.8 | (8.7) | 11.0 | (11.0) | 49.9 | (42.5) | 365.4 | (197.5) | 825.5 | (257.5) |  |  |

| Phthalate metabolites | Taiwan | Detectable rate (%) | *n* | Min | | 5th | | 25th | | 50th | | 75th | | 95th | | Max | | *P*-value ^b^ | |
| --- | --- | --- | --- | --- | --- | --- | --- | --- | --- | --- | --- | --- | --- | --- | --- | --- | --- | --- | --- |
| MiBP | Northern Taiwan | 68.6 | 121 | ND | (ND) | ND | (ND) | ND | (ND) | 10.6 | (10.6) | 18.8 | (20.8) | 67.9 | (42.6) | 138.0 | (200.1) | <0.001 | (<0.001) |
|  | Central Taiwan | 46.3 | 54 | ND | (ND) | ND | (ND) | ND | (ND) | 0.7 | (0.7) | 12.3 | (15.4) | 83.7 | (64.5) | 129.7 | (286.6) |  |  |
|  | Southern Taiwan | 97.1 | 102 | ND | (ND) | 4.0 | (2.8) | 9.9 | (8.2) | 18.4 | (18.4) | 29.8 | (26.7) | 84.2 | (86.5) | 115.0 | (149.4) |  |  |
|  | Eastern Taiwan | 67.7 | 65 | ND | (ND) | ND | (ND) | ND | (ND) | 6.7 | (6.7) | 15.6 | (16.6) | 49.4 | (36.1) | 101.9 | (141.7) |  |  |
|  | Remote | 68.9 | 45 | ND | (ND) | ND | (ND) | ND | (ND) | 8.9 | (8.9) | 14.8 | (13.7) | 59.1 | (28.6) | 75.8 | (70.2) |  |  |
| MEP | Northern Taiwan | 95.9 | 121 | ND | (ND) | 1.7 | (2.5) | 7.7 | (5.4) | 14.5 | (14.5) | 33.3 | (32.4) | 175.4 | (214.6) | 2376.0 | (3286.0) | <0.001 | (0.001) |
|  | Central Taiwan | 85.2 | 54 | ND | (ND) | ND | (ND) | 4.0 | (2.5) | 11.5 | (11.5) | 24.4 | (27.7) | 146.5 | (153.8) | 444.9 | (213.6) |  |  |
|  | Southern Taiwan | 93.1 | 102 | ND | (ND) | ND | (ND) | 8.8 | (8.0) | 17.4 | (17.4) | 39.2 | (30.3) | 154.6 | (230.8) | 4675.1 | (2571.3) |  |  |
|  | Eastern Taiwan | 90.8 | 65 | ND | (ND) | ND | (ND) | 7.3 | (6.0) | 20.2 | (20.2) | 34.7 | (31.8) | 293.8 | (157.0) | 1919.0 | (1017.1) |  |  |
|  | Remote | 84.4 | 45 | ND | (ND) | ND | (ND) | 2.8 | (3.0) | 8.8 | (8.8) | 13.1 | (13.3) | 46.4 | (67.0) | 412.0 | (626.4) |  |  |
| MiNP | Northern Taiwan | 5.8 | 121 | ND | (ND) | ND | (ND) | ND | (ND) | ND | (ND) | ND | (ND) | 4.3 | (2.4) | 43.4 | (49.5) | 0.36 | (0.001) |
|  | Central Taiwan | 11.1 | 54 | ND | (ND) | ND | (ND) | ND | (ND) | ND | (ND) | ND | (ND) | 12.9 | (7.0) | 16.8 | (22.2) |  |  |
|  | Southern Taiwan | 21.6 | 102 | ND | (ND) | ND | (ND) | ND | (ND) | ND | (ND) | ND | (ND) | 8.2 | (10.0) | 39.7 | (24.8) |  |  |
|  | Eastern Taiwan | 3.1 | 65 | ND | (ND) | ND | (ND) | ND | (ND) | ND | (ND) | ND | (ND) | ND | (ND) | 9.0 | (3.3) |  |  |
|  | Remote | 13.3 | 45 | ND | (ND) | ND | (ND) | ND | (ND) | ND | (ND) | ND | (ND) | 4.5 | (3.7) | 6.8 | (8.5) |  |  |
| MBzP | Northern Taiwan | 25.6 | 121 | ND | (ND) | ND | (ND) | ND | (ND) | 0.3 | (0.3) | 1.2 | (2.1) | 8.0 | (4.6) | 10.0 | (8.5) | <0.001 | (<0.001) |
|  | Central Taiwan | 18.5 | 54 | ND | (ND) | ND | (ND) | ND | (ND) | 0.3 | (0.3) | 0.6 | (0.2) | 7.2 | (9.1) | 12.7 | (27.1) |  |  |
|  | Southern Taiwan | 35.3 | 102 | ND | (ND) | ND | (ND) | ND | (ND) | 0.3 | (0.3) | 2.7 | (3.2) | 11.8 | (11.2) | 75.9 | (18.4) |  |  |
|  | Eastern Taiwan | 13.8 | 65 | ND | (ND) | ND | (ND) | ND | (ND) | ND | (ND) | ND | (ND) | 3.8 | (6.3) | 43.7 | (15.7) |  |  |
|  | Remote | 2.2 | 45 | ND | (ND) | ND | (ND) | ND | (ND) | ND | (ND) | ND | (ND) | ND | (ND) | 2.6 | (2.2) |  |  |
| MMP | Northern Taiwan | 98.3 | 121 | ND | (ND) | 4.6 | (4.8) | 14.7 | (10.0) | 30.3 | (30.3) | 49.3 | (41.7) | 176.6 | (123.8) | 6529.6 | (7117.3) | 0.001 | (<0.001) |
|  | Central Taiwan | 92.6 | 54 | ND | (ND) | ND | (ND) | 16.8 | (16.0) | 40.2 | (40.2) | 89.6 | (63.8) | 336.3 | (290.9) | 538.5 | (780.9) |  |  |
|  | Southern Taiwan | 99 | 102 | ND | (ND) | 9.2 | (5.9) | 19.7 | (15.5) | 34.7 | (34.7) | 70.9 | (66.2) | 253.5 | (278.7) | 2854.2 | (924.3) |  |  |
|  | Eastern Taiwan | 96.9 | 65 | ND | (ND) | 4.1 | (2.4) | 9.8 | (7.3) | 23.0 | (23.0) | 54.8 | (60.3) | 294.5 | (325.8) | 865.6 | (1350.3) |  |  |
|  | Remote | 93.3 | 45 | ND | (ND) | ND | (ND) | 25.0 | (19.5) | 65.3 | (65.3) | 129.1 | (169.9) | 1103.2 | (910.9) | 6216.1 | (3660.6) |  |  |

^a^ Abbreviations are listed in the footnote of Table 2; unadjusted level of each phthalate metabolite was shown in the parentheses; ND: not detectable; Detectable rate=number of urine sample with level of each phthalate metabolite above detection limit/ all analyzed urine samples.

^b^ Kruskal Wallis test; p-value for unadjusted level of each phthalate metabolite was shown in the parentheses.
