# Supplementary material for: Age and Gender Differences in Urinary Levels of Eleven Phthalate Metabolites in General Taiwanese Population after a DEHP Episode
Source: PLoS One. 2015 Jul 24;10(7):e0133782. doi: 10.1371/journal.pone.0133782 (PMC4514596; doi:10.1371/journal.pone.0133782)
Supplement: S4 Table — (DOCX) [file pone.0133782.s004.docx]

**S4 Table.** **Number** ^a^ **of individuals who use phthalate-containing food-related plastic products, cosmetic and personal care products, medicine and pesticides.**

| Items | **Adults (N=290)** | **Minors (N=97)** |
| --- | --- | --- |
| Food container (plastics) ^b^ | 30 (10.3) | 19 (19.8) |
| Food preservation film ^c^ | 212 (73.1) | 71 (73.2) |
| Food preservation box ^c^ | 155 (53.4) | 46 (47.4) |
| Food preservation bag ^c^ | 221 (76.2) | 71 (73.2) |
| Body wash ^d^ | 126 (43.6) | 71 (73.2) |
| Lotion ^d^ | 84 (29.1) | 7 (7.2) |
| Perfume ^d^ | 6 (2.1) | 0 (0) |
| Nail polish ^d^ | 1 (0.3) | 0 (0) |
| Medicine ^e^ | 123 (42.4) | 13 (13.4) |
| Pesticide use ^f^ | 71 (24.5) | 27 (27.8) |

^a^ Data was presented as N (%);

| ^b^ Subjects who self-reported using plastic containers for food; |
| --- |
| ^c^ Subjects who self-reported using preservation films for storage in a refrigerator or heating in a microwave oven; |
| ^d^ Subjects who self-reported using it one time per day in the past month; |
| ^e^ Subjects who self-reported taking prescriptive medicine in the past month; |
| ^f^ Subjects who self-reported using pesticide at home. |
