# Supplementary material for: Age and Gender Differences in Urinary Levels of Eleven Phthalate Metabolites in General Taiwanese Population after a DEHP Episode
Source: PLoS One. 2015 Jul 24;10(7):e0133782. doi: 10.1371/journal.pone.0133782 (PMC4514596; doi:10.1371/journal.pone.0133782)
Supplement: S5 Table — (DOCX) [file pone.0133782.s005.docx]

**S5 Table**. **Differences in subject creatinine levels (mg/dl) by age and gender (N=387) in our study.**

| Subjects | *N* | Male | Female | *P*-value ^a^ |
| --- | --- | --- | --- | --- |
| All subjects | 387 | 102 (17.0~462) | 71.0 (9.0~301) | <0.001 |
| Minors | 97 | 112 (27.4-420) | 87.8 (21.0-229) | 0.002 |
| Adults | 290 | 96.0 (17.0-462) | 64.1 (9.0-301) | <0.001 |
| Sub-group ^b^ | 363 | 103 (30.2~297) | 72.8 (31.0~259) | <0.001 |
| Minors | 92 | 110 (49.0~297) | 89.3 (45.9~229) | 0.007 |
| Adults | 271 | 96.2 (30.3~296) | 82.0 (30.3~296) | <0.001 |

^a^ Median (range); Mann-Whitney U test.

^b^ 24 subjects with creatinine<30 or ≧300 mg/dl were excluded.
