# Supplementary material for: Age and Gender Differences in Urinary Levels of Eleven Phthalate Metabolites in General Taiwanese Population after a DEHP Episode
Source: PLoS One. 2015 Jul 24;10(7):e0133782. doi: 10.1371/journal.pone.0133782 (PMC4514596; doi:10.1371/journal.pone.0133782)
Supplement: S6 Table — (DOCX) [file pone.0133782.s006.docx]

**S6 Table. Pearson correlation coefficients among 11 urinary phthalate metabolites (creatinine-adjusted) in Taiwanese minors (n=97, upper right) and Taiwanese adults (n=290, age≧18 years old, lower left), and adjusted/ unadjusted creatinine levels (diagonal) for all subjects.**

| Phthalate  metabolites ^a^ | MMP | MEP | MiBP | MnBP | MBzP | MEHP | MEHHP | MEOHP | MECPP | MCMHP | MiNP | ΣDEHPm | ΣDBPm |
| --- | --- | --- | --- | --- | --- | --- | --- | --- | --- | --- | --- | --- | --- |
| MMP | 0.91*** | -0.043 | -0.057 | 0.055 | -0.123 | 0.137 | 0.138 | 0.215^*^ | 0.079 | 0.160 | 0.130 | 0.176^#^ | 0.015 |
| MEP | 0.040 | 0.94*** | 0.294^**^ | 0.086 | 0.184^#^ | -0.015 | 0.008 | 0.204^*^ | 0.062 | 0.257^*^ | -0.102 | 0.070 | 0.244^*^ |
| MiBP | -0.047 | 0.139^*^ | 0.96*** | 0.127 | 0.256^*^ | 0.144 | 0.099 | 0.172^#^ | 0.026 | 0.200^*^ | 0.079 | 0.186^#^ | 0.662^**^ |
| MnBP | 0.083 | 0.096 | 0.188^**^ | 0.94*** | 0.126 | 0.071 | 0.112 | 0.224^*^ | 0.080 | -0.006 | 0.004 | 0.158 | 0.636^**^ |
| MBzP | 0.075 | 0.066 | 0.082 | 0.100^#^ | 0.9*** | -0.006 | 0.195^#^ | 0.257^*^ | 0.241^*^ | 0.158 | 0.086 | 0.256^*^ | 0.275^**^ |
| MEHP | 0.110^#^ | -0.030 | 0.203^**^ | 0.145^*^ | 0.020 | 0.94*** | 0.309^**^ | 0.358^**^ | 0.149 | 0.225^*^ | 0.253^*^ | 0.483^**^ | 0.238^*^ |
| MEHHP | 0.205^**^ | 0.061 | 0.055 | 0.187^**^ | 0.160^**^ | 0.313^**^ | 0.83*** | 0.523^**^ | 0.784^**^ | 0.297^**^ | 0.083 | 0.782^**^ | 0.221^*^ |
| MEOHP | 0.209^**^ | 0.063 | 0.186^**^ | 0.254^**^ | 0.188^**^ | 0.202^**^ | 0.398^**^ | 0.87*** | 0.414^**^ | 0.543^**^ | 0.293^**^ | 0.802^**^ | 0.389^**^ |
| MECPP | 0.128^*^ | -0.002 | -0.081 | 0.104^#^ | 0.199^**^ | 0.134^*^ | 0.562^**^ | 0.193^**^ | 0.88*** | 0.262^**^ | -0.097 | 0.707^**^ | 0.165 |
| MCMHP | 0.136^*^ | 0.106^#^ | 0.110^#^ | 0.148^*^ | 0.147^*^ | 0.030 | 0.269^**^ | 0.509^**^ | 0.329^**^ | 0.94*** | 0.098 | 0.568^**^ | 0.146 |
| MiNP | 0.070 | 0.019 | 0.072 | 0.014 | 0.252^**^ | 0.089 | -0.005 | 0.107^#^ | 0.044 | 0.020 | 0.86*** | 0.273^**^ | 0.143 |
| ΣDEHPm ^b^ | 0.235^**^ | 0.068 | 0.146^*^ | 0.280^**^ | 0.317^**^ | 0.477^**^ | 0.802^**^ | 0.550^**^ | 0.675^**^ | 0.511^**^ | 0.158^**^ | 0.89*** | 0.348^**^ |
| ΣDBPm ^c^ | 0.025 | 0.190^**^ | 0.613^**^ | 0.772^**^ | 0.129^*^ | 0.217^**^ | 0.176^**^ | 0.330^**^ | 0.042 | 0.183^**^ | 0.057 | 0.278^**^ | 0.67*** |

^a^ Abbreviations are listed in the footnote of Table 2. The level of each phthalate metabolite was log-transformed; ^#^: *p*<0.10; ^*^: *p* <0.05; ^**^: *p* <0.01; ^***^: *p* <0.001;

^b^ ΣDEHPm= sum of five DEHP metabolites (MEHP, MEHHP, MEOHP, MECPP, MCMHP);

^c^ ΣDBPm= sum of two DBP metabolites (MnBP and MiBP).
